# Investigation of the compaction process of electrical machines magnetic circuits and its detrimental effect on magnetic performances

**DOI:** 10.1038/s41598-022-23634-7

**Published:** 2022-11-08

**Authors:** Hugo Helbling, Adrien Van Gorp, Abdelkader Benabou, Thierry Coorevits, Abdelmounaïm Tounzi, Walid Boughanmi, Daniel Laloy

**Affiliations:** 1 Univ. Lille, Arts et Metiers Institute of Technology, Centrale Lille, Junia, ULR 2697 - L2EP, F-59000 Lille, France; 2 Arts & Metiers Institute of Technology, Mechanics, Surfaces and Materials Processing (MSMP), Lille, France; 3 JEUMONT Electric, 59460 Jeumont, France

**Keywords:** Electrical and electronic engineering, Magnetic properties and materials

## Abstract

The manufacturing processes of electrical machines may lead to significant degradation of the magnetic properties of their magnetic core (stator, rotor) performances and, as a consequence, to a decrease of their energy efficiency. While the effects of some processes (cutting, welding …) are widely discussed in the literature, this is not the case with the compaction process although it is systematically used to maintain the assembly of electrical steel sheets that compose the magnetic circuits. In addition to the conventional one, a specific compaction process exists for high-power electrical machines. After an introduction, the paper firstly deals with the two studied processes (conventional, specific). Then, an experimental mock-up to study the impact of the two configurations on the magnetic properties (iron losses, normal magnetization curve) is presented. This mock-up is the first, in the literature, that allows to study the effect of a controlled compaction mechanical stress on magnetic properties. Obtained results in both configurations highlight a magneto-mechanical effect that is not reported in the literature where these effects are commonly considered following in-plane mechanical stresses. This paper presents a magneto-mechanical model, taking into account the compaction stress effect, as well as a modelling protocol to model the effect of 3D mechanical stress on magnetic properties, which has never been done in the literature.

## Introduction

In the context of energy transition, increasing the energy efficiency of electrical machines is a key point. This involves reducing the losses and in particular the iron loss contribution associated to the magnetic circuits. The latter are difficult to quantify accurately and there still exist variable and significant differences between the iron losses estimated by the manufacturer during the design stage and those measured on the machine once manufactured. These differences are noticeably due to the electrical steel magnetic properties degradation following the manufacturing process. The significance and type of this effect are strongly related to the complexity of the microstructure of the magnetic materials as well as to their strong mechanical and thermal couplings^1–3^. In particular, it is shown in the literature that the manufacturing processes of the magnetic circuits of electrical machines can lead to significant degradation on their magnetic performances (iron losses, normal magnetization curve)^4,5^. While the effects of some manufacturing processes (such as cutting^6^, welding^7^, shrink-fitting^8^, …) are widely studied in the literature, the compaction process remains rarely studied.

The magnetic cores of electrical machines are mainly composed of stacked electrical steel sheets. In order to maintain the lamination assembly, a compaction mechanical effort is applied along the laminations thickness direction. Usually maintained by a dedicated system (clamping screws, bars welded to yokes …), this compaction effort implies the presence of mechanical stresses within the laminations. Depending on the configuration of the application of the compaction force, the mechanical stresses can be three-dimensional (in the plane or along the thickness direction of the sheets).

According to the literature, mechanical stress applied in the plane of electrical steel sheets has a strong influence on their magnetic properties^1^: this can improve or degrade them depending on the nature of the stress (compressive stress or tensile stress), the level of stress and its direction with regard to the magnetic flux direction. However, for mechanical stress applied in the thickness direction of the lamination, few papers are found in the literature and the presented results vary significantly from one study to another^9–11^. Obviously, in this direction, one has to only consider compressive stress because tensile stress has no physical application and is against the objective of the compaction process. It is shown in^10^ that the application of an axial compressive stress of up to 24MPa induces an improvement (up to 8MPa) and then a degradation (beyond 8MPa) of the magnetic properties of a toroidal core composed of a single sheet. In^11^, the authors study the effects of localized stress, up to 10MPa, in the thickness direction of a rectangular magnetic sheet: the result is a degradation of the magnetic properties. Moreover, the observed degradations on the magnetic properties are similar, considering a magnetic flux along the rolling direction, for a stress of 0.5MPa and for a stress of 10MPa. In^9^, a dedicated ring core tester is developed to study the effect of axial stress on a single ring core sample. Contrary to what is observed in^10^, a degradation of the magnetic properties is first observed (up to 10MPa) and then a gradual improvement appears up to 30MPa.

These significant variations in the results can be explained by several factors. Firstly, the possible presence of mechanical stresses in the plane of the samples, due to potential imperfection of the applied normal stress, is not verified. However, we have seen, according to the literature, that in-plane stresses can have a significant effect on magnetic properties. Noticeably in^9^, mechanical Finite Element (FE) simulations show an important difference between the distribution, within the studied electrical steel sheet, of the stress applied in the axial direction and the distribution of the Von Mises stress (a scalar equivalent stress considering the 3D mechanical stress distribution), thus suggesting the presence of non-negligible stresses in the plane of the sample. Then, since the studies were conducted on magnetic circuits composed of very few laminations (usually only one), these are in proportion more sensitive to the friction at the interface with device used for the compaction force application, which is more likely to induce stress in the plane of the laminations. Finally, the studied geometry undoubtedly has an influence on the distribution of mechanical stresses as it is shown in^12^. In general, the fact that existing works do not make it possible to control and/or know the 3D mechanical stress distribution makes it impossible to conclude on the effect, on magnetic properties, of mechanical stresses applied in the thickness direction or the development of generalizable and predictable magneto-mechanical models modelling the effect of compaction process on magnetic properties.

In that context, this paper proposes to study the effect of two different configurations of compaction process on the magnetic properties of an electrical steel stack: a conventional configuration with a homogeneous compaction and a specific one, which is mostly employed to manufacture high-power electrical machines, exhibiting heterogeneous compaction pressure. These two configurations will be firstly presented as well as the experimental mock-up developed to study their effect on magnetic properties of electrical steel laminations. Then, experimental results will be presented and discussed to move on to the modelling phase. For this part, mechanical FE simulations were performed. Moreover, magneto-mechanical models and a protocol for modeling the effect of 3D mechanical stresses on magnetic properties are described. The experimental results and those given by the modelling approach are then compared and discussed. Finally, conclusions and perspectives of this work will be given.

## Experimental mock-up

### Compaction configurations

**Figure 1:**
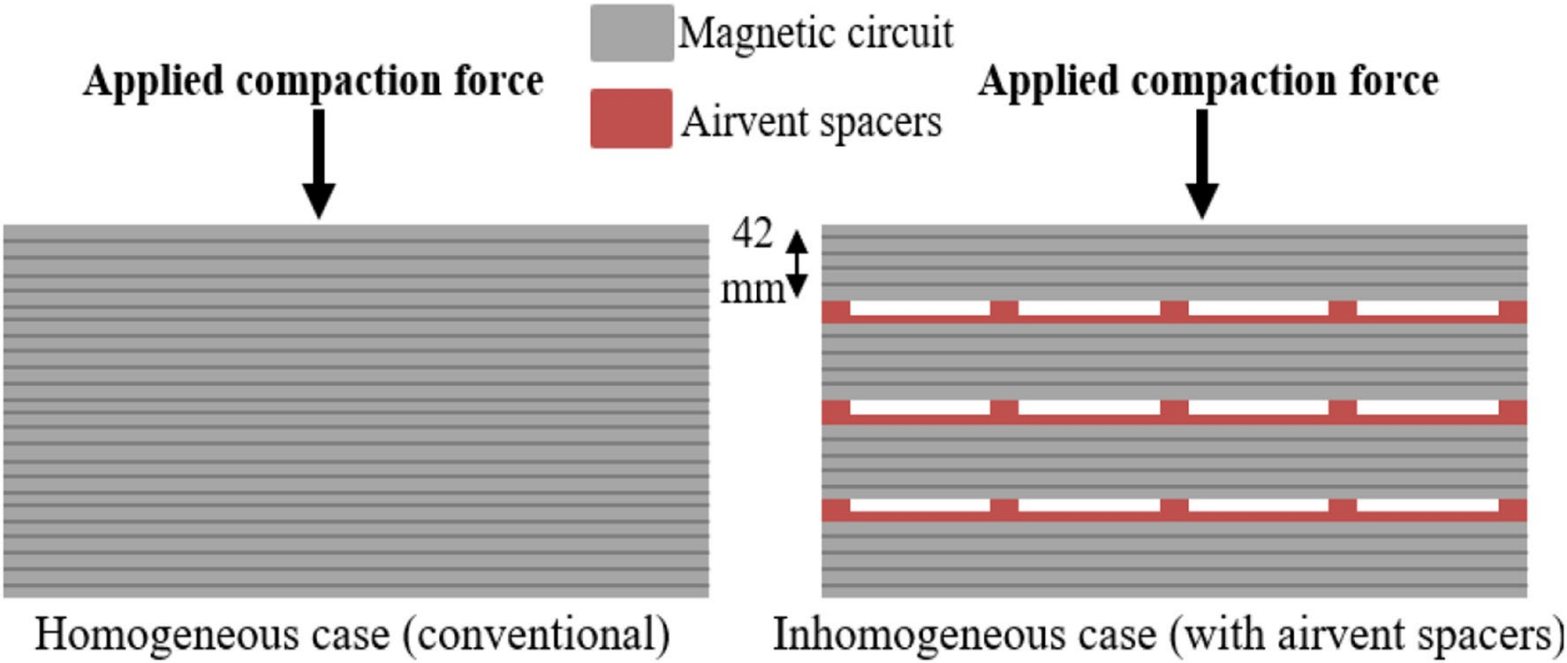
The two studied configurations.

**Figure 2:**
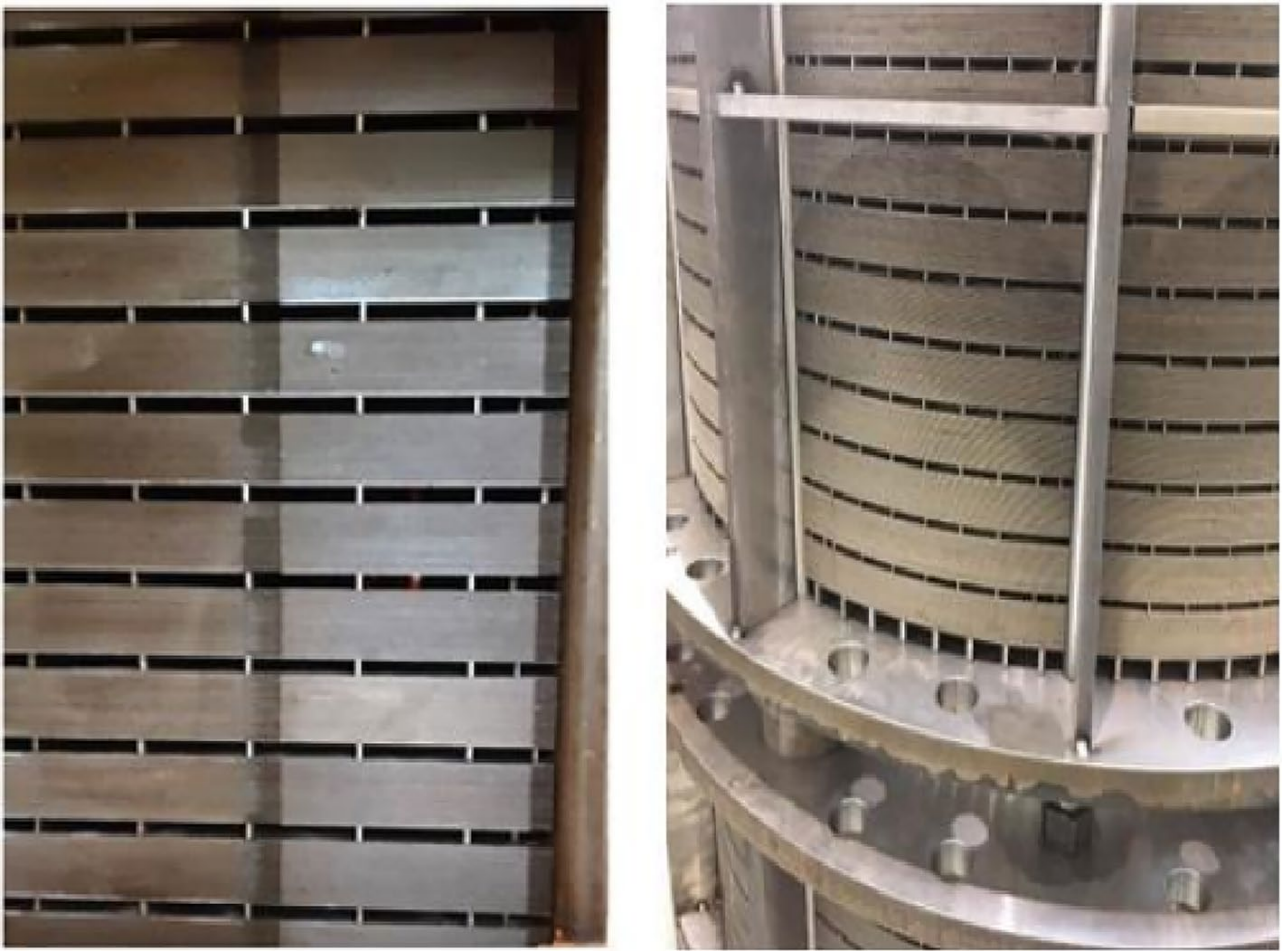
View of two industrial stators (with airvent spacers).

The conventional compaction process involves pressing a single stack of electrical steel sheets that makes up the stator or the rotor. In the case of large alternators, the magnetic circuit is usually divided into several stacks separated by interlayer sheets composed of airvent spacers. The objective of this configuration is to facilitate the cooling of the machine by thermal convection. The conventional configuration will be considered in the following as the homogeneous case (the compaction force is applied homogeneously over the whole circuit). The second configuration, with airvent spacers, will be denominated as the inhomogeneous case (the presence of airvent spacers generates localized mechanical stresses). Both configurations are presented in Fig. 1. In the inhomogeneous case, the industrial height of laminations stack is typically of few centimeters. As illustration, photos of two industrial stators with airvent spacers are given in Fig. 2.

Generally, the global compaction stress (over the whole magnetic core) has the same value for the homogeneous and inhomogeneous cases (about 1 MPa). In the inhomogeneous case, the value of the localized mechanical stress under the airvent spacers is between 5 MPa and 20 MPa, depending on the number of airvent spacers and their geometry.

### Design and fabrication

To characterize the magnetic properties (iron losses, normal magnetization curve) of a magnetic core in the two presented configurations, a dedicated magnetic mock up is developed. First, concerning the magnetic circuit, the selected electrical steel is a conventional material with a lamination thickness of 0.65 mm (grade M600-65A) commonly used in high-power electrical machines. Moreover, for the experimental investigations, a toroidal geometry is preferred because it is closer to the geometry of the electrical machine stator cores. Therefore, several rings were cut from the raw laminations by Wire Electrical Discharge Machining (WEDM) that is known as a non-degrading cutting method for the magnetic properties^13^. The final height of the magnetic core, made from stacked rings, is 42 mm. All the characteristics of the used magnetic circuit are presented in Table 1.

**Table 1:** Magnetic core characteristics.

Laminations grade	Insulation	Cutting method
M600-65A	Coated (Alkophos method)	Wire Electrical Discharge Machining (WEDM)

Outer diameter (OD)	Inner diameter (OD)	Height
100 mm	86 mm	42 mm

**Figure 3:**
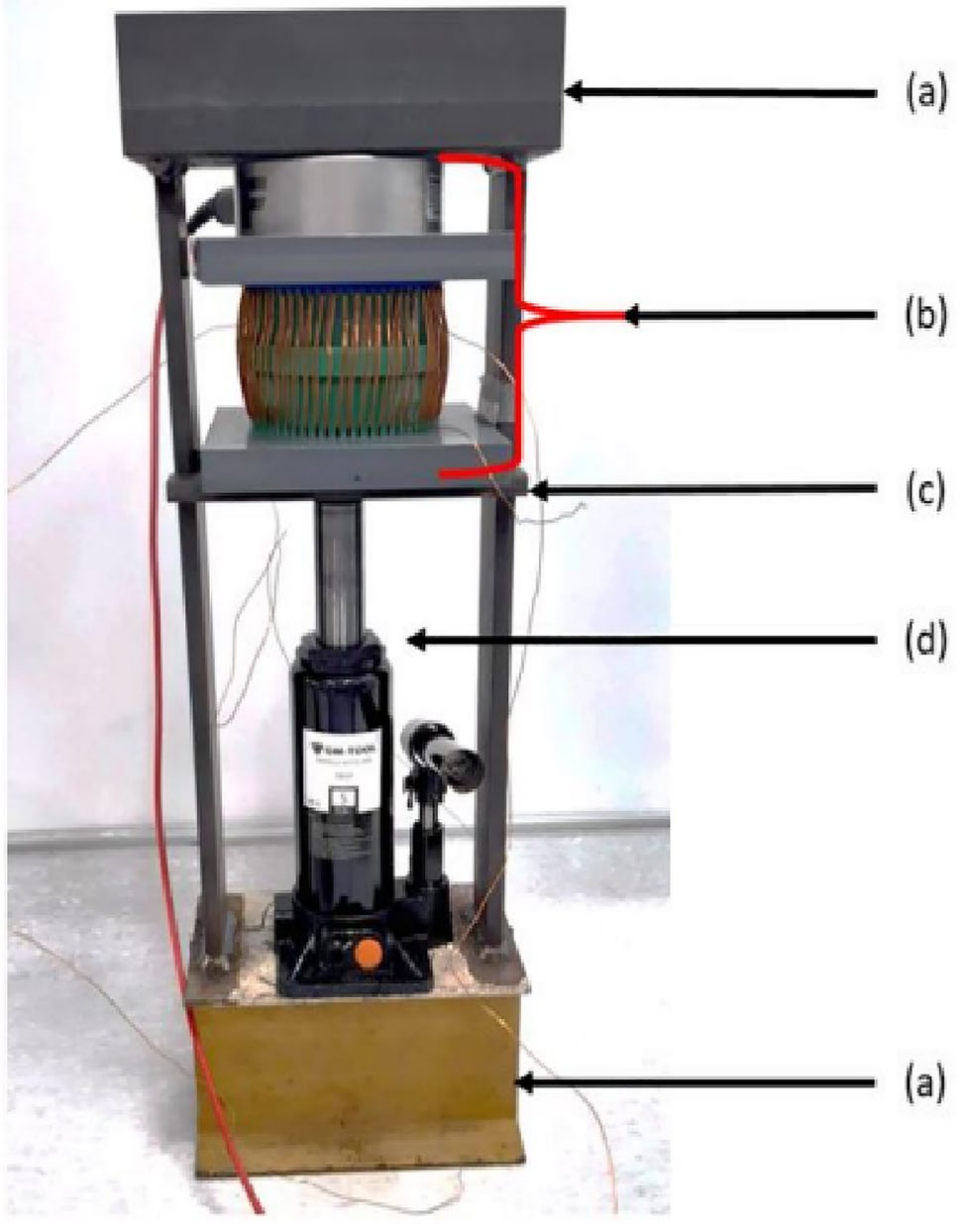
Global mock-up; (**a**) fixed structure of the press; (**b**) see Fig. 4; (**c**) movable steel plate; (**d**) hydraulic jack.

**Figure 4:**
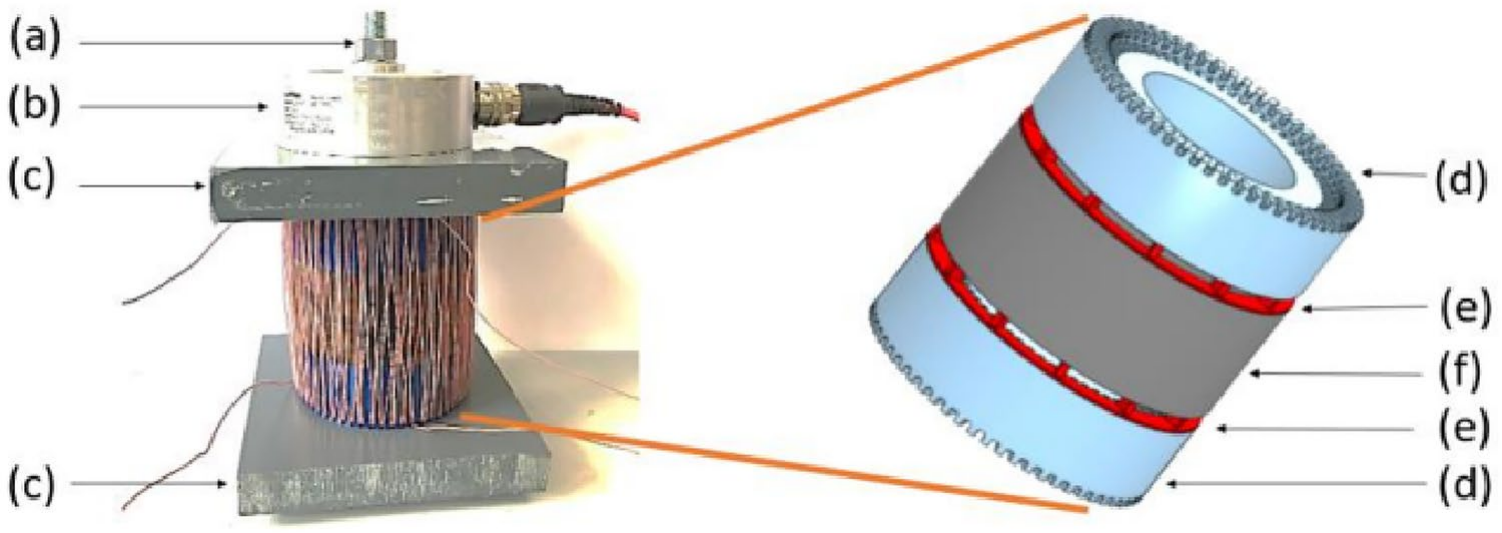
Zoom of the mock-up; (**a**) screw; (**b**) force sensor; (**c**) PVC plate; (**d**) winding frame; (**e**) airvent spacers support; (**f**) laminated magnetic circuit.

**Figure 5:**
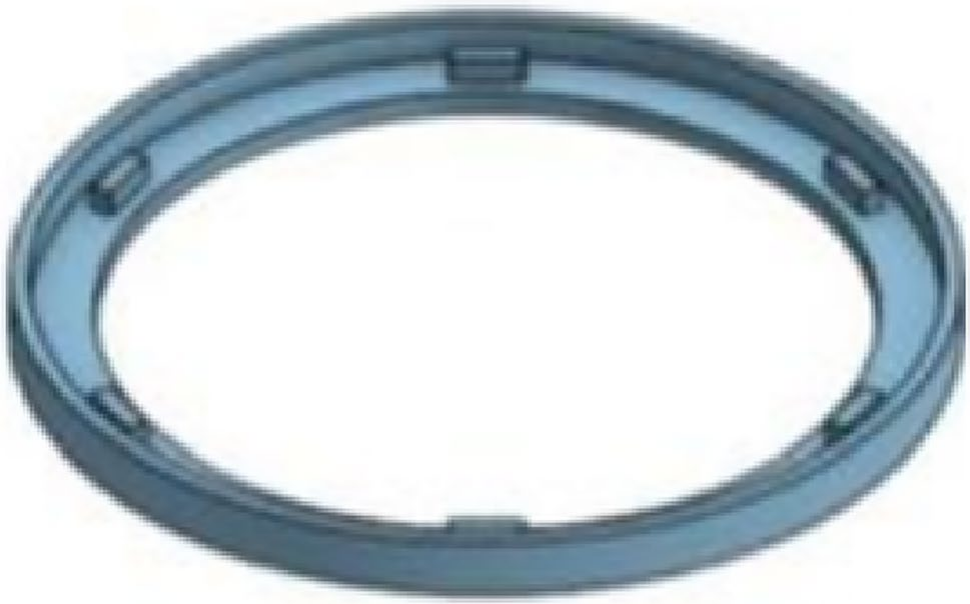
Airvent spacers support.

The global mock-up is shown in Fig. 3, a zoom of the part composed by the magnetic circuit, the winding supports, the PVC plates, the screw and the force sensor is given in Fig. 4. Finally, the support with the spacers is presented in Fig. 5.

Two winding supports, Fig. 4d, are placed on either side of the magnetic core, Fig. 4f. This makes possible to insert the primary and secondary windings while keeping the ability to apply the mechanical stress. The material used for the winding supports is Lab1000 that is amagnetic and electrically nonconductive. PVC plates, Fig. 4c, are also placed on both sides of the winding supports to promote the stress distribution homogeneity. These plates are also machined to have a good flatness. The whole is hold tight together with a force sensor, Fig. 4b, itself solidarized and centered with the fixed structure of the press, Fig. 3a. This latter is composed of metal elements welded together and designed to support the mechanical levels of stress that will be applied by a hydraulic jack, Fig. 3d. For the inhomogeneous case, airvent spacers supports, Fig. 5, are machined with the same material as the winding supports (amagnetic and electrically nonconductive). In this case, the airvent spacers supports are placed between the magnetic circuit and the winding supports, Fig. 4. For the homogeneous case, these are simply removed. Note that in Fig. 5, there is an outer ring around the spacers to promote the alignment between the different components of the mock-up. However, in order to facilitate the visualization of the airvent spacers, this outer ring has been removed from the view in Fig. 4.

To validate the experimental mock-up, it is necessary to verify that, in the homogeneous case, the axial mechanical stress distribution is homogeneous within the magnetic core and that the mechanical stress distribution in the plane of the laminations is negligible in order to quantify only the effect of the normal mechanical stress on the magnetic properties.

First, mechanical Finite Element (FE) simulations were performed with the Abaqus Software^14^. According to the involved levels of mechanical stress, the mechanical behavior of all the materials will remain in the elastic region. The list of materials chosen for the FE simulations and their associated mechanical properties are given in Table 2.

**Table 2:** Mechanical properties used for mechanical FE simulations.

Part of the mock-up	Material	Young’s modulus (GPa)	Poisson’s ratio (–)
Force sensor	Aluminium	70	0.34
PVC plate	PVC	3	0.40
Winding support	Lab1000	5.9	0.35
Magnetic laminations	Steel	211	0.29

The friction coefficients between the different parts need to be determined because of a potential significant effect on the mechanical stress distribution. Indeed, the mechanical parameters being different for each material, the deformations will also be different. Without friction, each part moves freely without any induced mechanical stress. With friction, deformations will be more or less hindered and parasitic mechanical stresses may appear in the plane of the electrical steel sheets. However, these friction coefficients are difficult to estimate because they depend on many parameters, such as the surface state of each part. The chosen approach is therefore as follows: according to the data in the literature, mechanical FE simulations are carried out in extreme cases for the highest and lowest values of friction coefficients in order to assess the influence of these parameters on the mechanical stress distribution. In addition, a PTFE film is placed between the winding supports and the magnetic circuit to reduce friction. Simulations results show that the mechanical stress distribution within the magnetic circuit varies slighlty as a function of the friction coefficients. Therefore, the chosen friction coefficients are those commonly found in the literature and are given in Table 3.

**Table 3:** Chosen friction coefficients for mechanical FE simulations.

Materials interfaces	Chosen friction coefficients
Screw/force sensor	0.4
Force sensor/PVC plate	0.5
PVC plate/winding support	0.5
Winding support/lamination (Teflon)	0.05
Lamination/lamination	0.2

**Figure 6:**
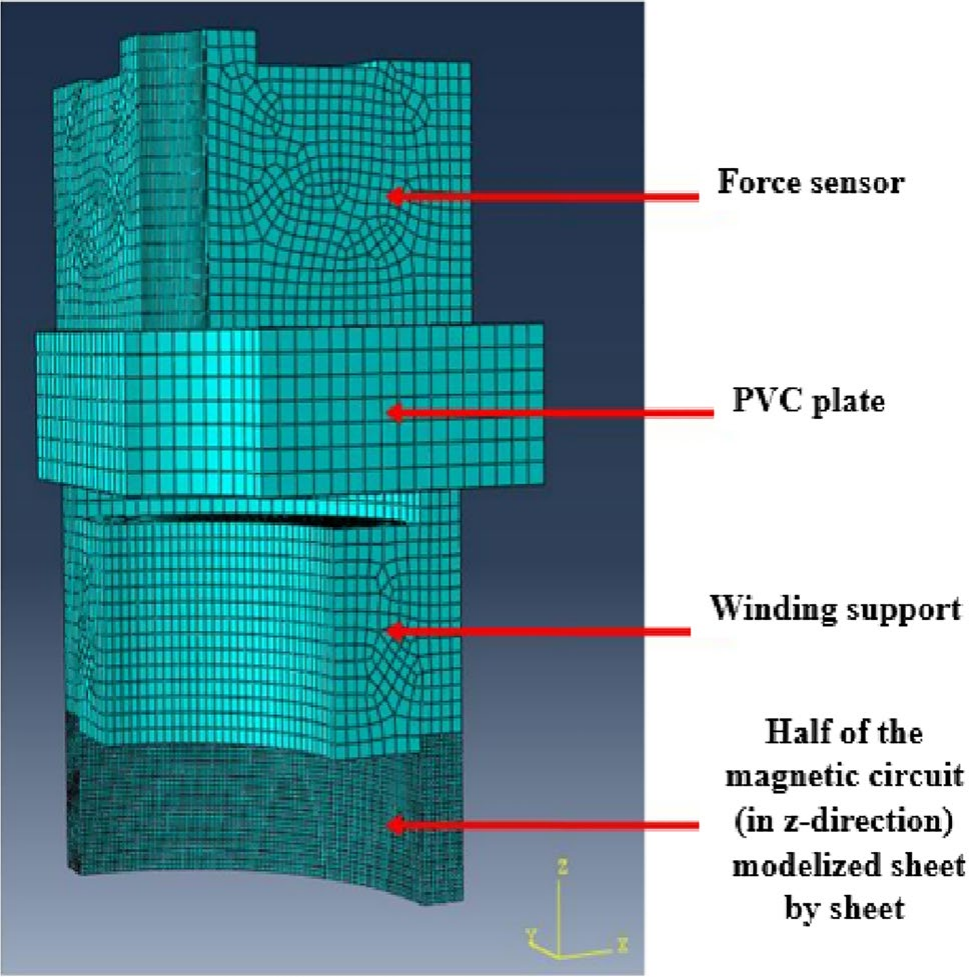
Mock-up modeled and meshed with Abaqus.

**Figure 7:**
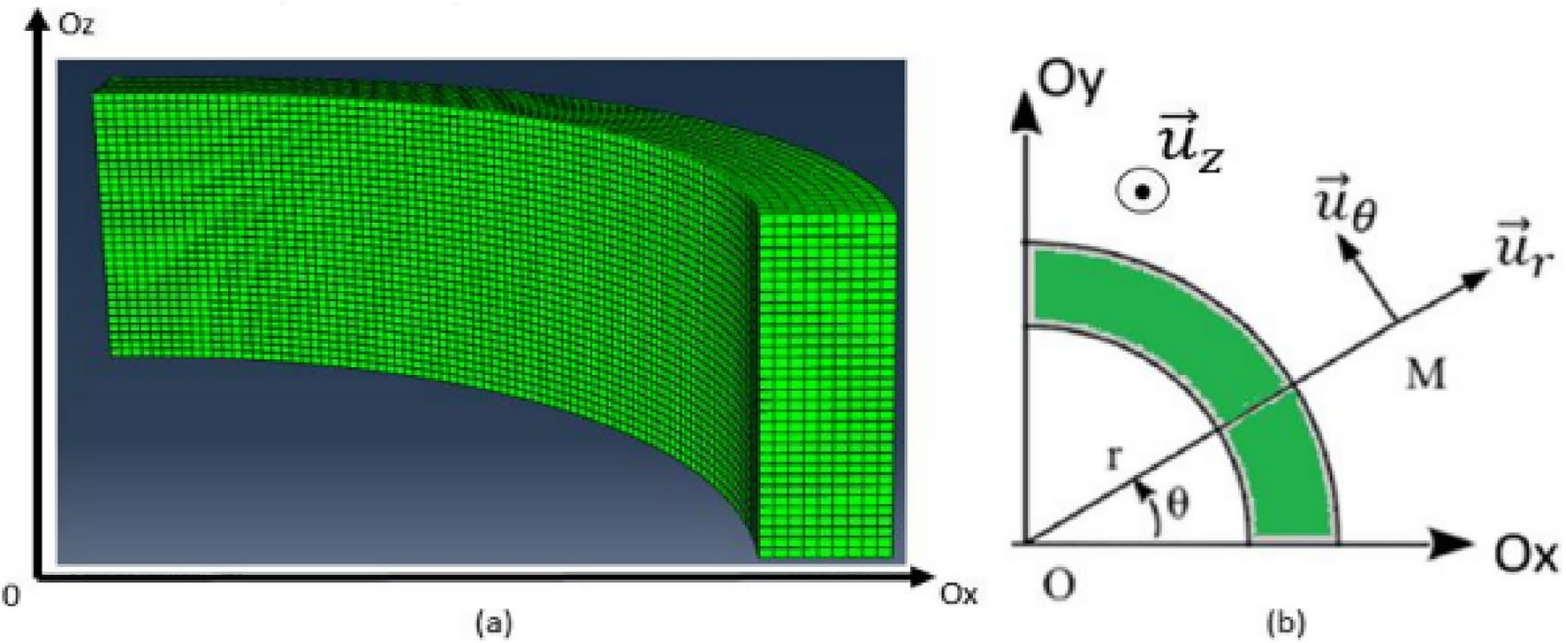
Coordinated system used cutting view (**a**) and top view (**b**) of the laminated magnetic circuit.

The Abaqus model and the coordinate system related to the magnetic circuit are respectively given in Figs. 6 and 7. Mechanical conditions of symmetries were taken into account whether axially or radially. In the following, the axial (in the thickness direction), orthoradial (in the magnetic flux direction, in the plane of the laminations) and radial (perpendicular to the magnetic flux direction, in the plane of the laminations) mechanical stresses will be respectively noted ${\upsigma }_{\mathrm{z}}$, ${\upsigma }_{\uptheta }$ and ${\upsigma }_{\mathrm{r}}$.

**Figure 8:**
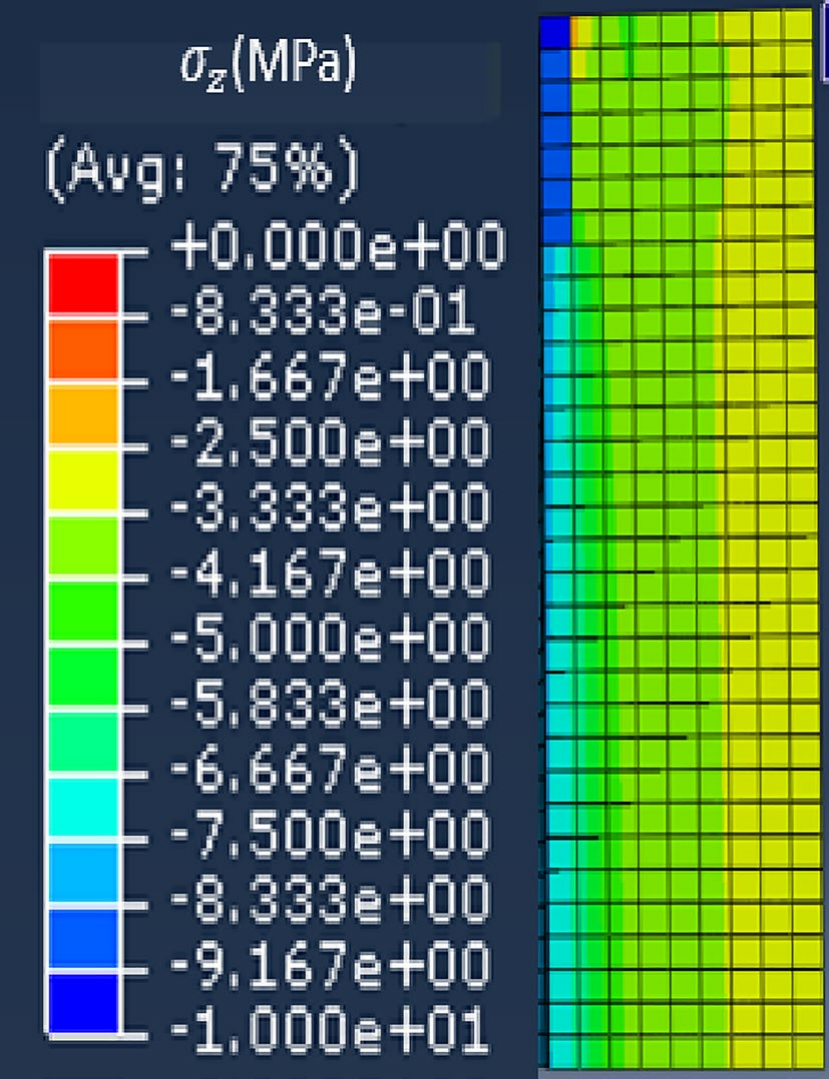
Axial mechanical stress distribution - Homogeneous case.

**Figure 9:**
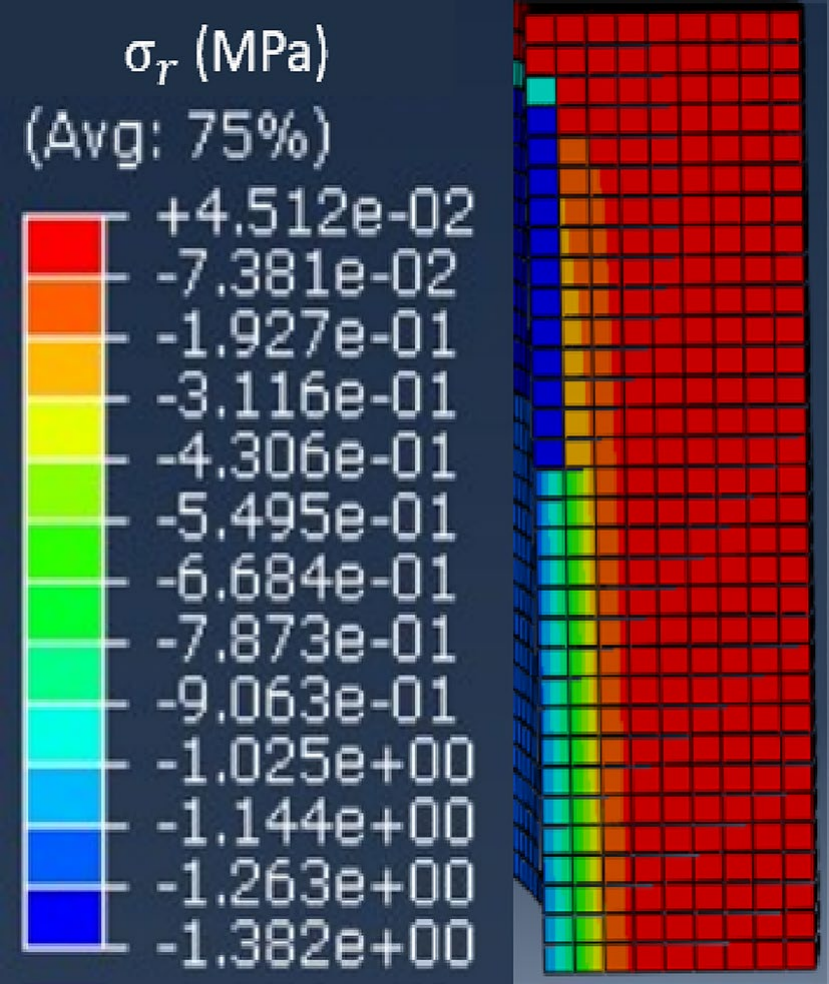
Radial mechanical stress distribution - Homogeneous case.

**Figure 10:**
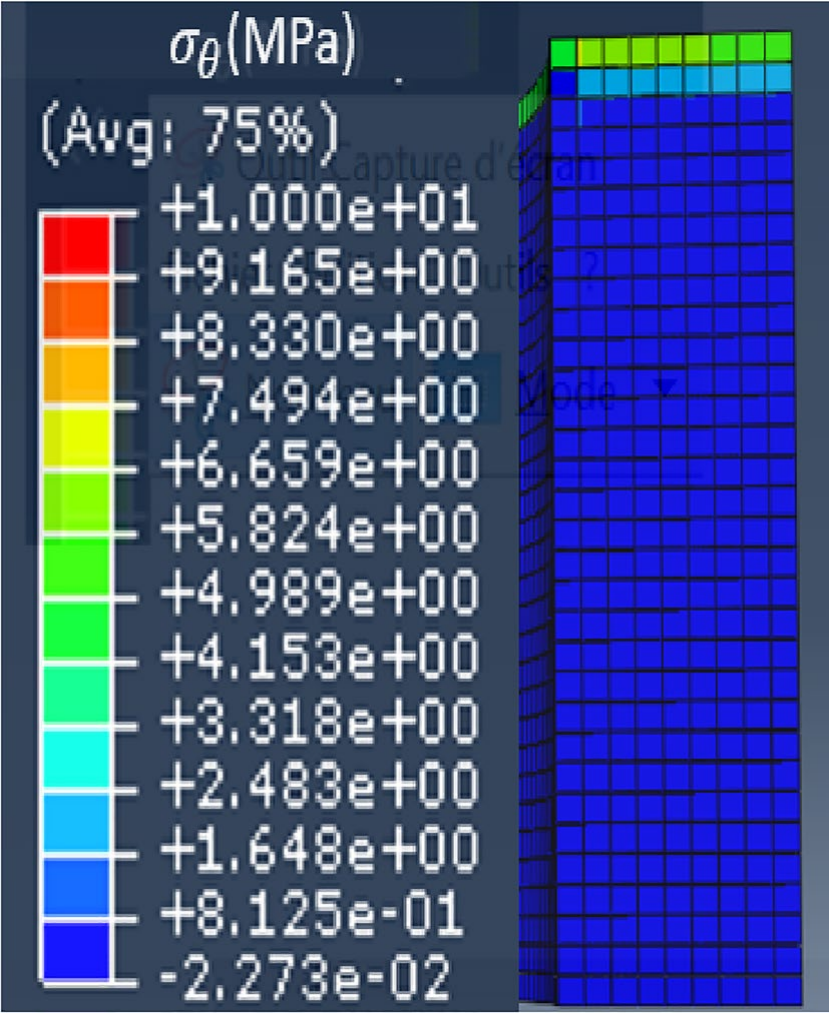
Orthoradial mechanical stress distribution - Homogeneous case.

Note that for the magnetic circuit, the height of one mesh element corresponds to the thickness of one electrical steel sheet. Moreover, the simulated homogeneous configuration corresponds to an applied theoretical axial mechanical stress of 4 MPa over the whole magnetic core. The mechanical stress distributions along ${\mathbf{u}}_{\mathbf{z}}$, ${\mathbf{u}}_{\mathbf{r}}$ and ${\mathbf{u}}_{{\varvec{\uptheta}}}$ are respectively given in Figs. 8, 9 and 10.

**Figure 11:**
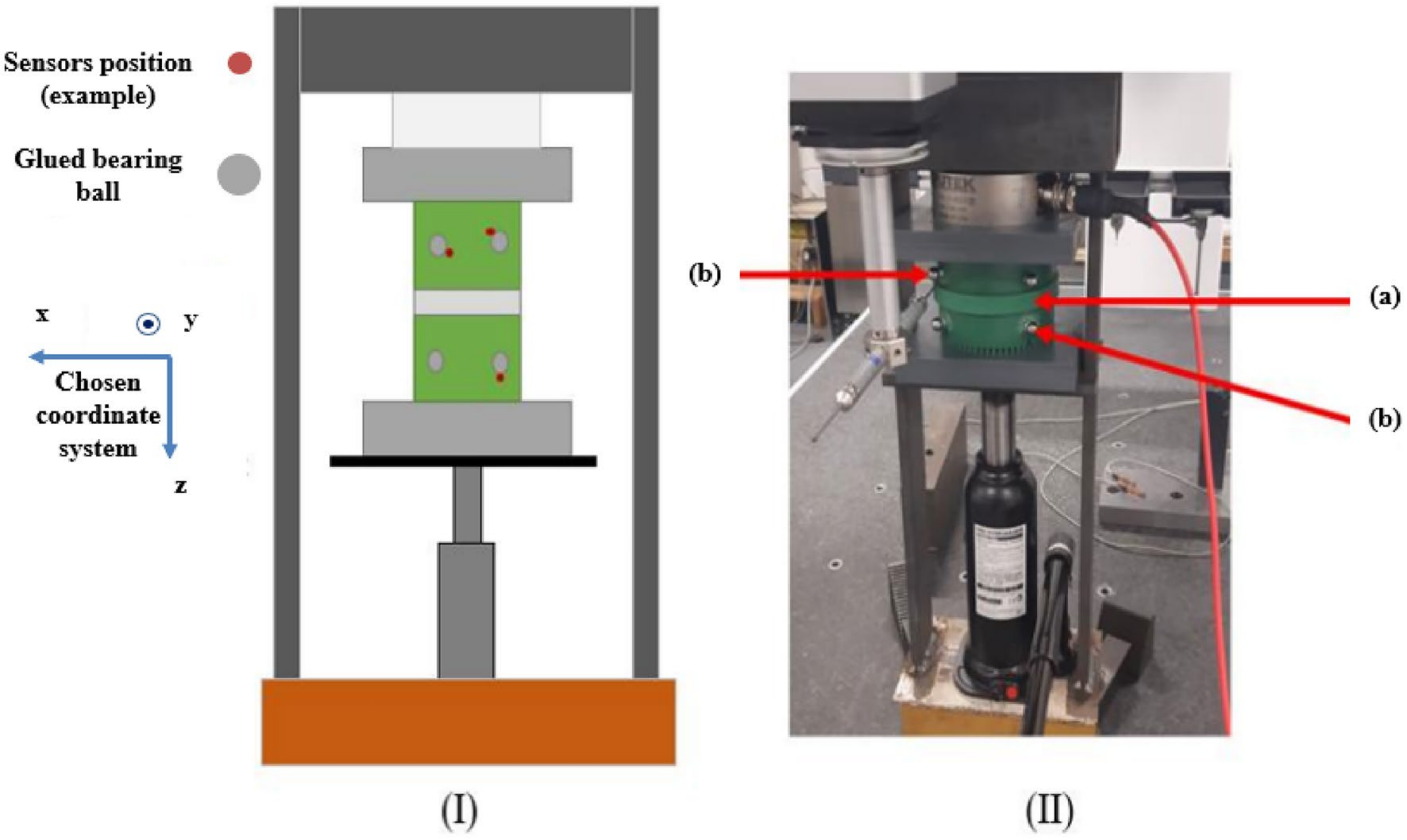
Principle of the metrology test; (**a**) centering ring; (**b**) bearing ball.

**Figure 12:**
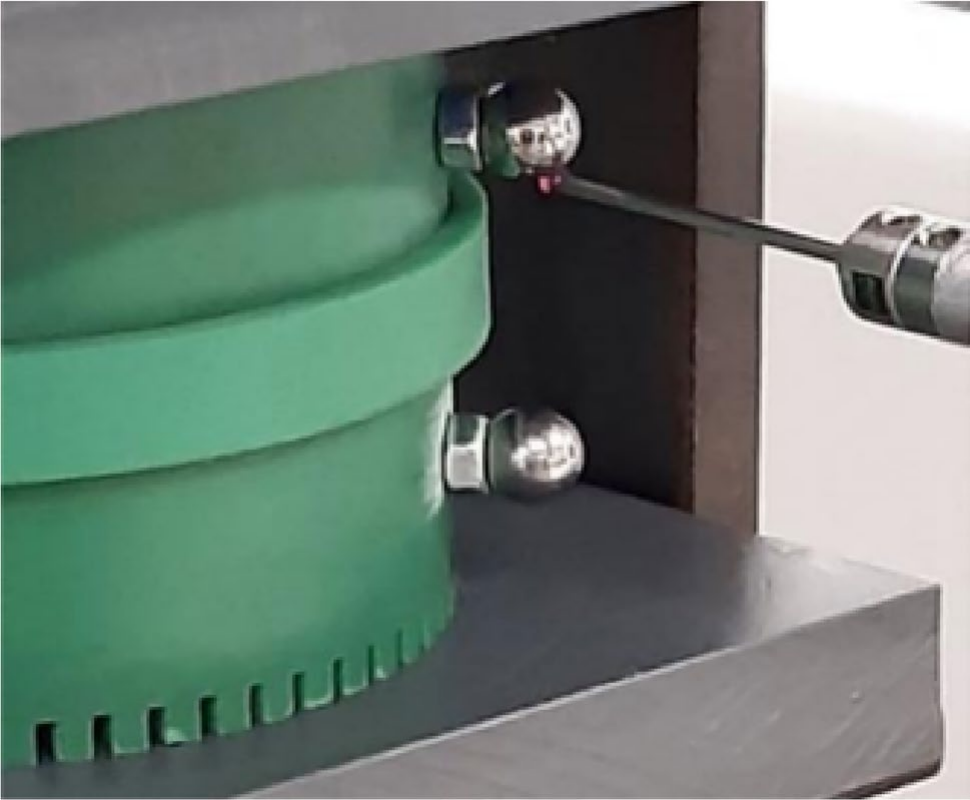
Probe measuring the position of a bearing ball.

First, results show that the axial stress ${\upsigma }_{\mathrm{z}}$ is globally homogeneous and of the order of − 4 MPa. Secondly, the induced stresses in the plane of the laminations (${\upsigma }_{\uptheta }$ and ${\upsigma }_{\mathrm{r}}$) are very low compared to the axial one. If the results of mechanical FE simulations seem to validate the mock-up, we must be able to confirm that we are in the same configuration experimentally as any misalignment or machining inaccuracy can lead to significant error in the stress distribution. For this, metrology tests were carried out, as illustrated in Fig. 11. The objective is to quantify the displacement of the two winding supports under compaction effort. As these are the parts that are the closest to the magnetic circuit, this will best reflect the mechanical behavior of the latter. First, eight bearing balls are glued to the outer diameter of the winding supports (four per support). Each ball is then measured on a high-precision coordinate measuring machine to determine its diameter (known) to confirm the measuring quality and its center position in the (**x**, **y**, **z**) coordinate system, Figs. 11 and 12. The measured diameters of the balls are very close (< 5 µm) to the theoretical diameter provided by the manufacturers. From the positions of the bearing balls, the position of each winding support can be determined by applying the least-squares method and, from these positions, the objective is to determine the kinematic torsor **T** to quantify the displacement of the supports. For this, winding supports are considered as non-deformable solids. The six components of the torsor **T**, defined at the center of each support, (1), noted (u, v, w) and (α, β, γ), represent respectively the translation and rotation of the winding support according to the three axes **x**, **y** and **z**. The indices *T* and *B* correspond respectively to the top and bottom winding support.

**Figure 13:**
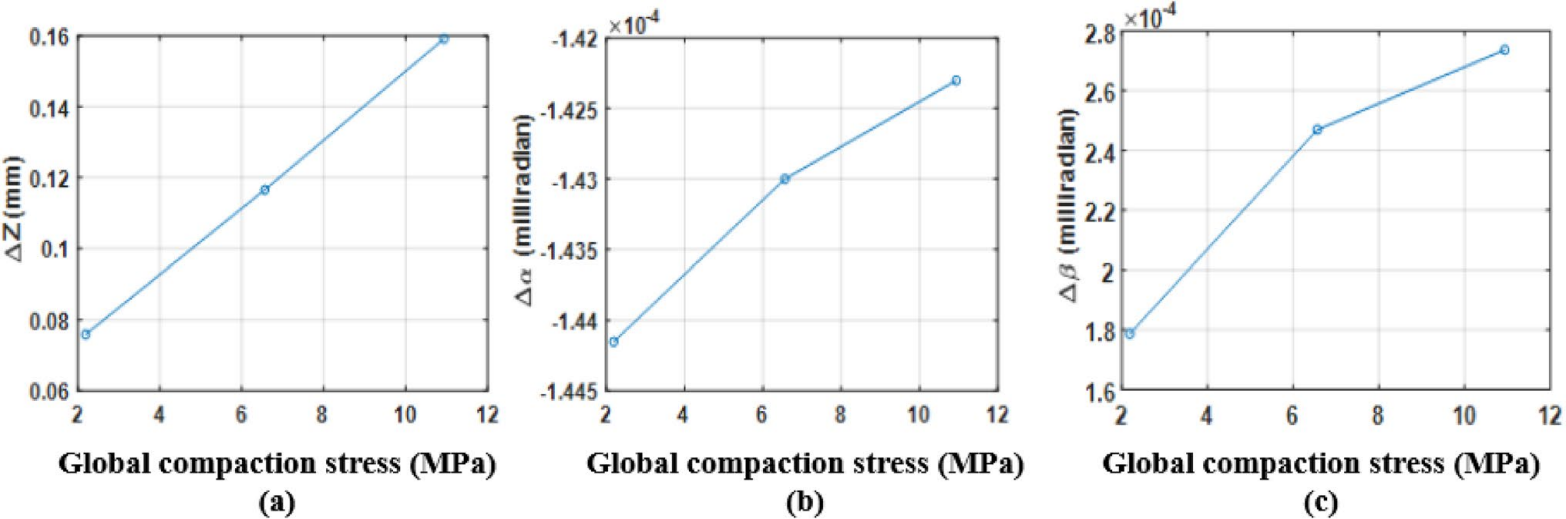
Evolution of **Δ** torsor components; (**a**) Δz; (**b**) Δα; (**c**) Δβ.

The goal is to determine the six components of each support and for each compaction level. To address this issue, the hypothesis of small displacements is first made (very small deformations of the magnetic core). Then, the torsor of each support is calculated, from a matrix formulation of the problem, thanks to the least-squares method which allows to obtain the torsor **Δ** that represents the difference between **T**_*T*_ and **T**_*B*_, (2). The quantities Δα and Δβ are the ones of interest because they correspond to the difference in rotation of the two supports around **x** and **y** axes and therefore are an image of the heterogeneity of the compaction stress distribution with regard to the lamination plane. If they are constant with the compression load, the distribution stays perfectly uniform. The idea is to determine the proportion of Δz that can be attributed to rotations Δα and Δβ for each magnetic core point. In practice, the measured Δz variation is 83.35 µm, Fig. 13a. By definition, a rotation of one milliradian induces a height variation of one millimeter over a length of one meter. Considering that the outer diameter of the magnetic circuit and the winding supports is 100 mm, the values of Δα and Δβ, Fig. 13b,c, lead respectively to a height variation of 1.9 µm and 9.5 µm that represents 2.3% and 11.4% of Δz variation at the diameter. In the worst case, this represents 14% of Δz variation. It can therefore be considered that the compaction pressure is evenly distributed at ±7%. The experimental mock-up is therefore considered as valid if the impact on the magnetic properties is higher than 10% for a 11MPa compaction pressure with a quite proportional evolution for other loads.

1 $$\mathbf{T}=\left\{\begin{array}{cc}\begin{array}{c}\mathrm{u}\\ \mathrm{v}\\ \mathrm{w}\end{array}& \begin{array}{c}\mathrm{\alpha }\\\upbeta \\\upgamma \end{array}\end{array}\right\}$$

2 $${\varvec{\Delta}}=\left\{\begin{array}{cc}\begin{array}{c}{\mathrm{u}}_{T}-{\mathrm{u}}_{B}\\ {\mathrm{v}}_{T}-{\mathrm{v}}_{B}\\ {\mathrm{w}}_{T}-{\mathrm{w}}_{B}\end{array}& \begin{array}{c}{\mathrm{\alpha }}_{T}-{\mathrm{\alpha }}_{B}\\ {\upbeta }_{T}-{\upbeta }_{B}\\ {\upgamma }_{T}-{\upgamma }_{B}\end{array}\end{array}\right\}=\left\{\begin{array}{cc}\begin{array}{c}\Delta \mathrm{u}\\ \Delta \mathrm{v}\\ \Delta \mathrm{w}\end{array}& \begin{array}{c}\Delta \mathrm{\alpha }\\ \Delta\upbeta \\ \Delta\upgamma \end{array}\end{array}\right\}$$

## Experimental results

### Homogeneous case

The magnetic core presented in Table 1 is studied in terms of its magnetic properties evolution as a function of the compaction stress. The experimentally applied compaction stress levels vary from 0MPa to 20MPa. All the magnetic measurements presented in the rest of this paper are performed on the MPG200D equipment from Brockhaus Measurements in accordance with the standard IEC 60404-4^15^ that relies on the flux metric method to determine the magnetic properties. Measurements are carried out over a frequency range from 5 Hz to 300 Hz and for peak magnetic flux density ranging from 0.1 to 1.6 T. Finally, the measured magnetic flux density is corrected by considering the compensation of the air flux due to the non-negligible height of the winding supports. To synthetize the measured B-H loops, in the following we will consider the normal magnetization curve that is obtained from the extrema B_max_-H_max_ of the centered hysteresis loops.

**Figure 14:**
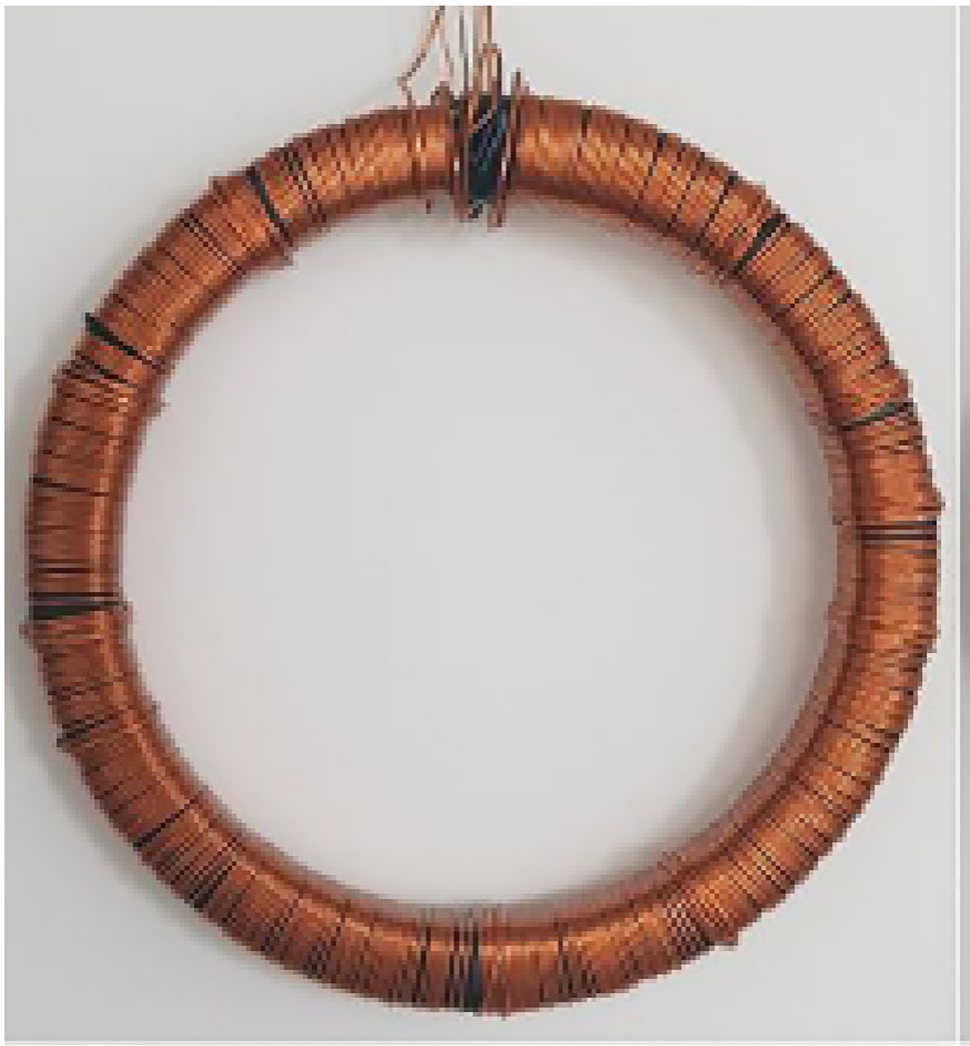
Reference magnetic circuit.

**Figure 15:**
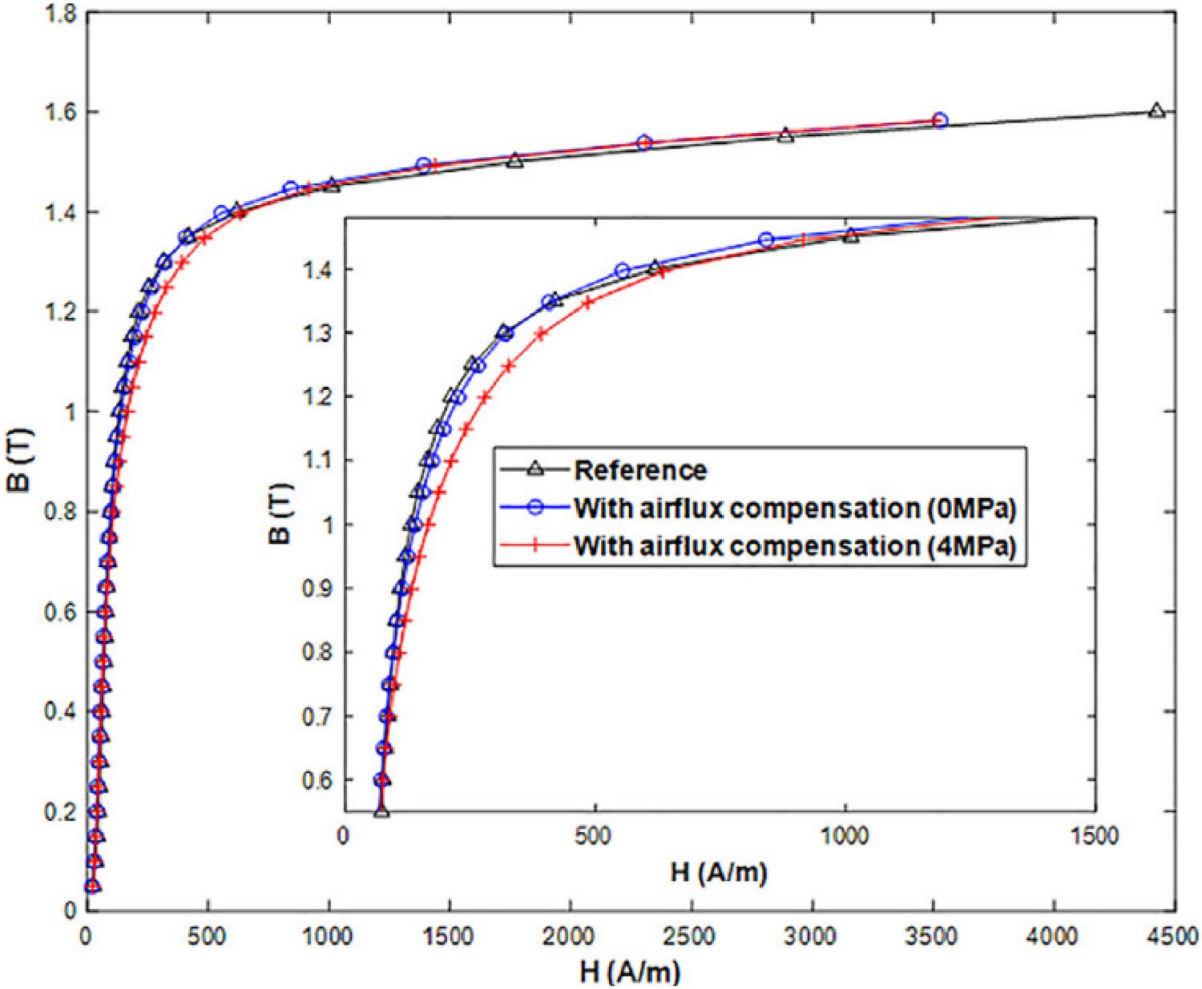
Validation of the air flux compensation method.

As example, and to validate the air flux correction, the results obtained with the mock-up with air flux compensation are compared to those given by a reference circuit, strictly identical but conventionally wound, Fig. 14. The obtained results, Fig. 15, show, on the one hand, that the reference normal magnetization curve and the one with air flux compensation (without compaction stress) are very close and, on the other hand, that the differences between them are negligible with regards to the effect of a homogeneous compaction at a level of 4 MPa. Finally, several measurements were performed to estimate the repeatability on the experimental mock-up: the obtained repeatability is then less than 1%.

**Figure 16:**
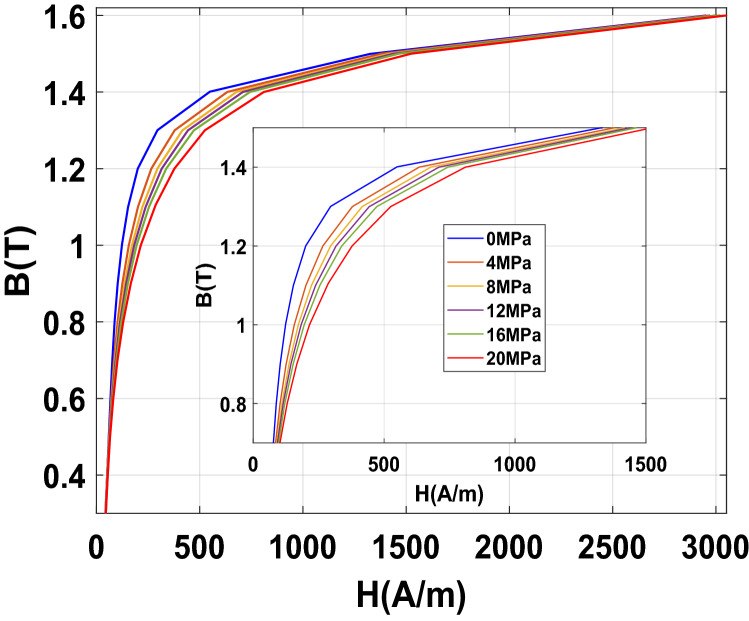
Normal magnetization curves 50 Hz - Homogeneous case.

**Figure 17:**
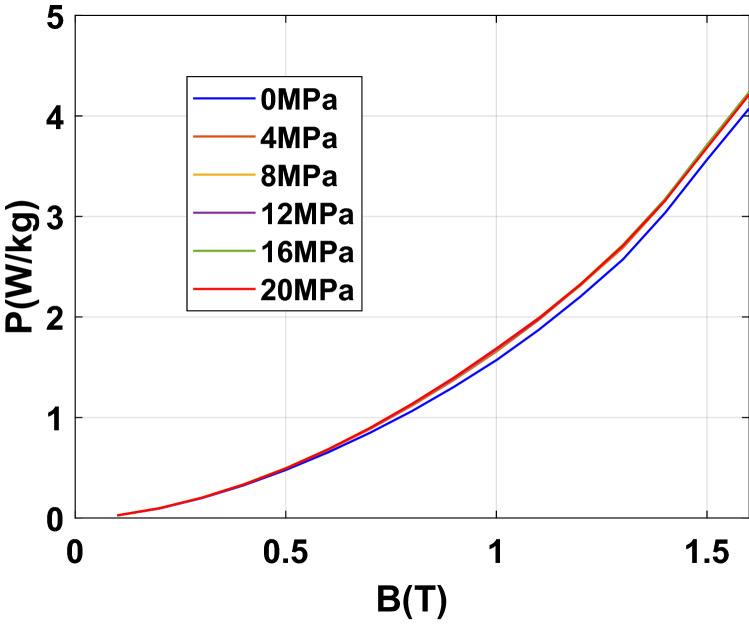
Iron losses 50 Hz - Homogeneous case.

**Figure 18:**
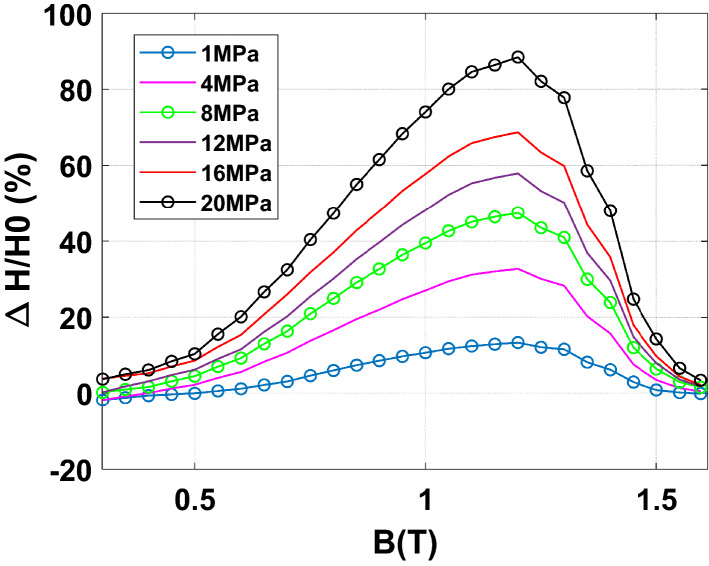
Relative difference on magnetic field due to compaction 50 Hz - Homogeneous case.

**Figure 19:**
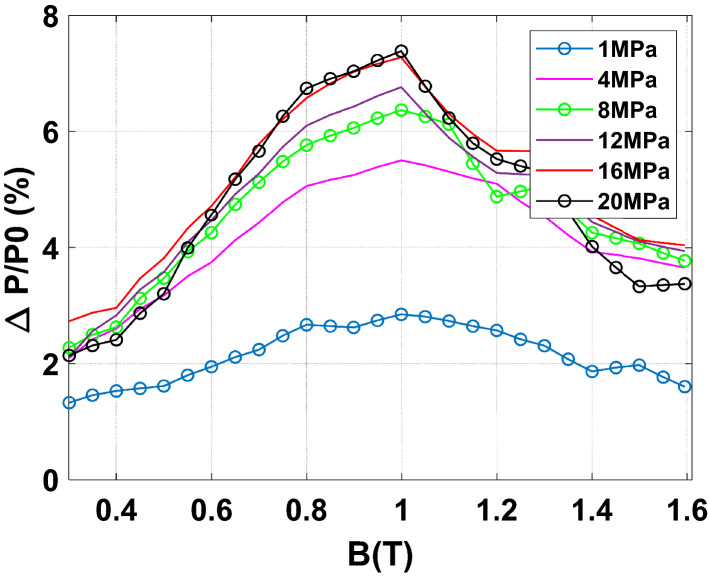
Relative difference on iron losses due to compaction 50 Hz - Homogeneous case.

To emphasize the effect of an increasing homogeneous compaction stress level, the normal magnetization curves and iron losses at 50 Hz are given, respectively, in Figs. 16 and 17. Results show a significant effect of the compaction stress on the normal magnetization curve mainly in the saturation knee. Moreover, for the iron losses, a limited increase is visible with the compaction stress: a small increase is directly observed at 4 MPa and beyond this threshold the iron losses remain quite unchanged. To better quantify these effects, they are expressed in terms of relative variation compared to the case without stress denoted as ${\mathrm{P}}_{0}$ for the losses and ${\mathrm{H}}_{0}$ for the magnetic field. These relative variations of losses $\Delta \mathrm{P}/{\mathrm{P}}_{0}$ (%) and magnetic field $\Delta \mathrm{H}/{\mathrm{H}}_{0}$ (%) are determined for each level of applied stress depending on the magnetic flux density level. The relative variations of the magnetic field and iron losses are given, respectively, in Figs. 18 and 19. The conclusions are similar with a significant and gradual degradation effect on the normal magnetization curve and a much more limited effect that saturates for iron losses, in the considered range of applied stress. It is not at all common, according to the literature, to observe such a significant degradation on the normal magnetization curve accompanied with a limited effect on iron losses, especially if one compares with the effects of mechanical stresses applied in the plane of the electrical steel sheets^1^. These observations will be discussed in the dedicated section of the paper.

**Figure 20:**
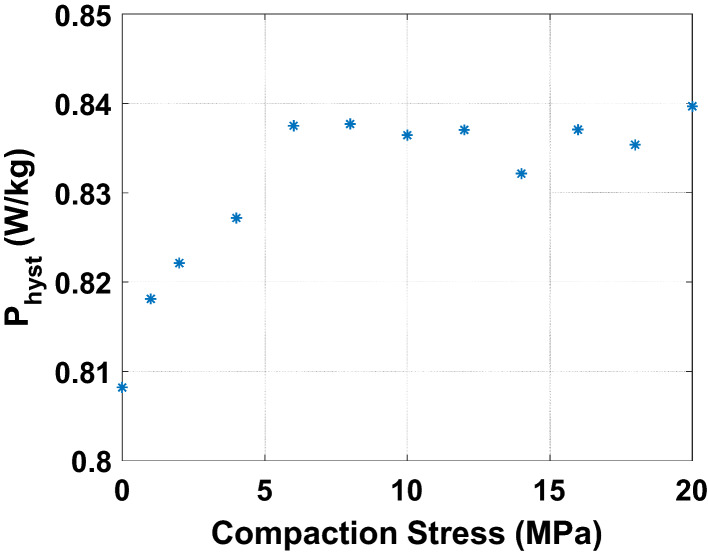
Hysteresis loss evolution as a function of compaction stress for 1 T at 50 Hz - Homogeneous case.

**Figure 21:**
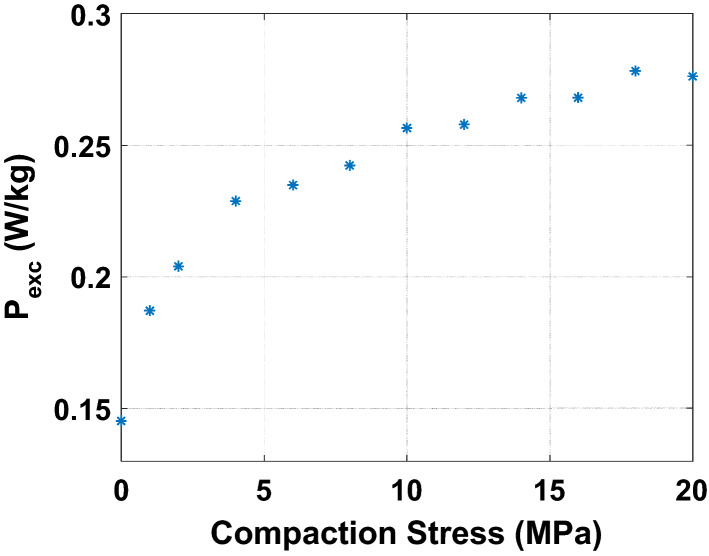
Excess loss evolution as a function of compaction stress for 1 T at 50 Hz - Homogeneous case.

A further analysis of these experimental results consists in decomposing the losses into their main physical contributions. In^16^, G. Bertotti proposed a decomposition of iron losses in three terms: hysteresis losses ${\mathrm{P}}_{\mathrm{hyst}}$ (static), classical losses ${\mathrm{P}}_{\mathrm{class}}$ (dynamic) and excess losses ${\mathrm{P}}_{\mathrm{exc}}$ (dynamic). The sum of these three terms constitutes the total iron losses. For an electrical steel sheet of thickness d and electrical conductivity ${\upsigma }_{\mathrm{e}}$ subjected to sinusoidal magnetic flux density of frequency f and peak value ${\mathrm{B}}_{\mathrm{max}}$, the classical losses are written as follows: ${\mathrm{P}}_{\mathrm{class}}=\frac{\uppi^{2}}{6}\cdot {\upsigma }_{\mathrm{e}}\cdot \mathrm{d}^{2}\cdot ({\mathrm{B}}_{\mathrm{max}}\cdot \mathrm{f})^{2}$. The hypothesis is made that these are not impacted (for the considered compaction stress range) by compaction for two reasons. First, considering the Young’s modulus E of the lamination, the applied mechanical stress ${\upsigma }_{\mathrm{m}}$ and the Hooke’s law, the thickness variation $\Delta \mathrm{d}=\frac{\mathrm{E}}{{\upsigma }_{\mathrm{m}}}$ is less than 0.1%. Moreover, measurements of the electrical conductivity, using the four needles technique, were also performed on a rectangular single sheet which was submitted to different compaction stress levels (using the experimental mock-up presented in Fig. 3). Results show that the electrical conductivity is not affected by the level of the considered compaction stress levels. Therefore, only the hysteresis and excess losses are potentially impacted. First, the classical losses are fitted (using experimental data without stress) according to their analytical expression. Then, the hysteresis losses are determined by an extrapolation of the total energy loss at 0 Hz and the excess losses by a power balance (${\mathrm{P}}_{\mathrm{exc}}={\mathrm{P}}_{\mathrm{tot}}-{\mathrm{P}}_{\mathrm{hyst}}-{\mathrm{P}}_{\mathrm{class}}$). The evolutions of hysteresis losses and excess losses as a function of the compaction stress are given in Figs. 20 and 21, for 1 T peak magnetic flux density at 50 Hz. The global tendency is an increase of the static and dynamic losses with the compaction stress. However, this increase is mostly observed for low levels of compaction stress before exhibiting stabilization at higher levels of compaction. These results are consistent with the measurements performed on the total iron losses presented in Fig. 17 and 19. Before continuing to discuss these results, the next step is to experimentally evaluate the effect of inhomogeneous compaction, i.e. in the presence of airvent spacers, on the magnetic properties.

### Inhomogeneous case

In the inhomogeneous case, airvent spacers support are placed between the magnetic core and the winding frames (Fig. 5). The mechanical compressive strength of the Lab1000 material (used for the airvent spacers and winding supports) is of about 110 MPa. In the homogeneous case, a global compaction stress of 4 MPa corresponds to the magnetic circuit surface that represents the contact area with the winding frame. In the inhomogeneous case, a global compaction stress of 4 MPa implies significant localized compressive stress under the airvent spacers. Indeed, in this case, the contact area with the winding frames is the total airvent spacers area that represent approximatively 5% of the magnetic circuit surface. Therefore, the localized compressive stresses are of the order of 80 MPa. The followed experimental protocol is the same as for the homogeneous case. The goal is to compare the evolution of magnetic properties due to the compaction process in the homogeneous and inhomogeneous cases, for global axial compaction pressure ranging from 0 MPa to 4 MPa.

**Figure 22:**
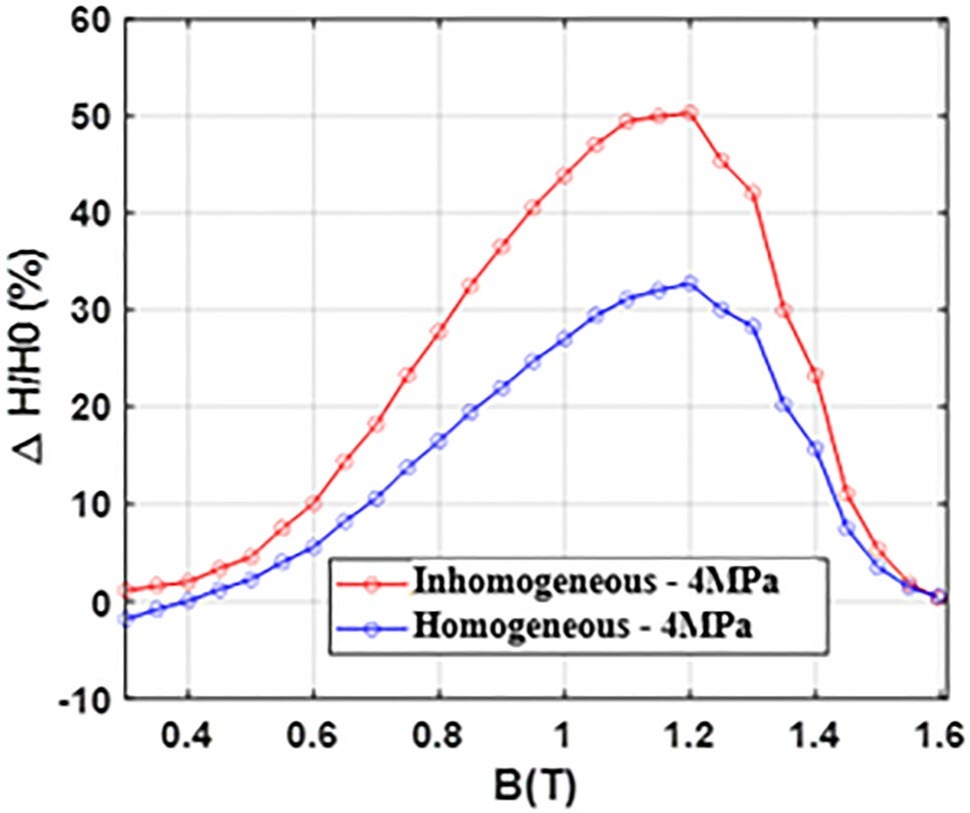
Relative evolution of magnetic field - Homogeneous and inhomogeneous cases comparison.

**Figure 23:**
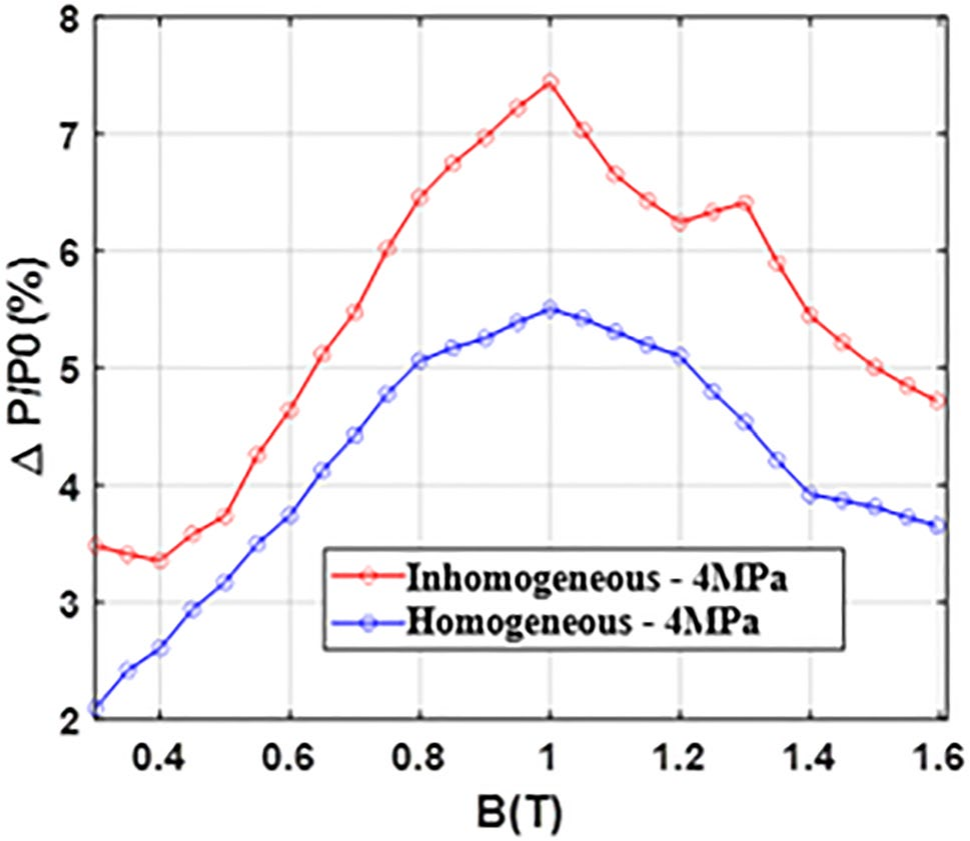
Relative evolution of iron losses - Homogeneous and inhomogeneous cases comparison.

The relative evolutions of the magnetic field and iron losses, in the homogeneous and inhomogeneous case, are reported in Figs. 22 and 23. These results highlight a key point: for a given overall compaction pressure of 4 MPa, the inhomogeneous case is more degrading than the homogeneous case with a maximum increase in the magnetic field of 50% (against 32% for the homogeneous case) and a maximum increase in iron losses of 7.5% (against 5.5% for the homogeneous case). In addition, as this was already observed in Figs. 18 and 19, the magnetic field is significantly impacted unlike iron losses which exhibit a limited increase.

### Discussion

**Figure 24:**
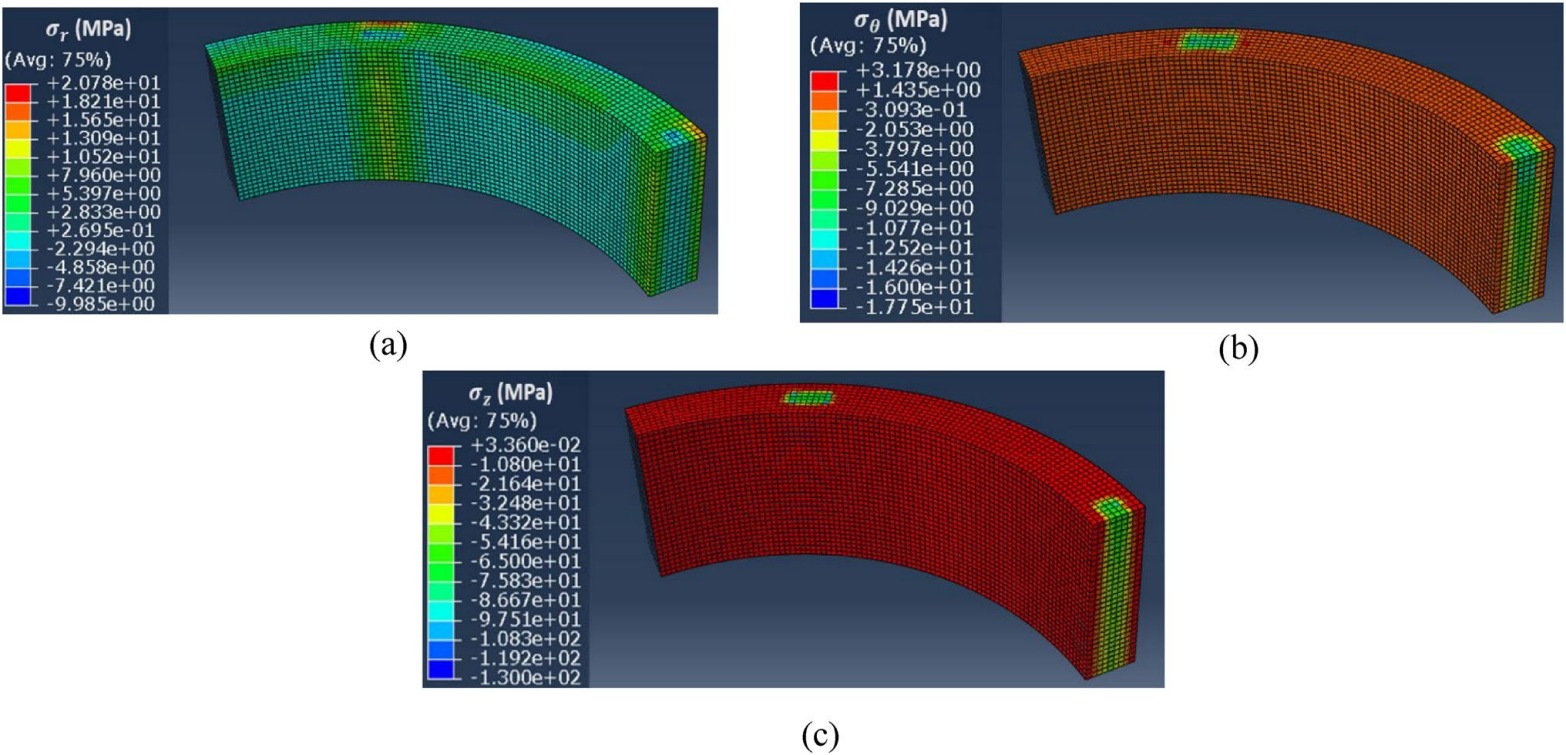
Mechanical stress distribution for the inhomogeneous case (4 MPa). (**a**) Radial (${\mathbf{u}}_{\mathbf{r}}$**)**, (**b**) Orthoradial (${\mathbf{u}}_{{\varvec{\uptheta}}}$**)** and (**c**) Axial (${\mathbf{u}}_{\mathbf{z}}$**).**

The objective is to understand the observed differences in terms of magnetic properties modifications between the homogeneous case and the inhomogeneous case. For this, mechanical FE simulations were performed in Abaqus for the inhomogeneous case while considering the mechanical parameters presented in Tables 2 and 3. Using the same coordinate system presented in Fig. 7, the mechanical stress distributions along the main directions ${\mathbf{u}}_{\mathbf{r}}$, ${\mathbf{u}}_{{\varvec{\uptheta}}}$ and ${\mathbf{u}}_{\mathbf{z}}$ are respectively given in Fig. 24a–c.

The first main result, Fig. 24a,b, is that the presence of airvent spacers implies, when applying a compaction effort, the appearance of noticeable mechanical stresses in the plane of the electrical steel sheets. According to the literature^1,17^, mechanical stresses in the plan have a significant effect on magnetic properties. These effects are described in Table 4, for the stress ranges given by the Abaqus simulations, in terms of qualitative impact on the magnetic properties.

**Table 4:** Effect of mechanical stress applied in the plane of electrical steel sheet on its magnetic properties^1,17^.

	Parallel to flux direction**	Perpendicular to flux direction**
Tensile stress	Improvement*	Degradation
Compressive stress	Degradation	Improvement*

*Up to a few tens of MPa, then a degradation is observed with higher stress levels.

**For an identical level of stress, stresses along the magnetic flux direction are quantitavely more impactful than those perpendicular^1^.

In our case, the direction parallel to the magnetic flux is ${\mathbf{u}}_{{\varvec{\uptheta}}}$ and the perpendicular direction (in the plane of laminations) is ${\mathbf{u}}_{\mathbf{r}}$. First, Fig. 24b shows that the mechanical stress along ${\mathbf{u}}_{{\varvec{\uptheta}}}$ is mainly localized under the airvent spacers along the height of the magnetic core with a compression level of about − 9 MPa, which will significantly degrade the magnetic properties (increase in iron losses, decrease in magnetic permeability). Second, in Fig. 24a, the mechanical stress along ${\mathbf{u}}_{\mathbf{r}}$ is divided into tensile stress area (green) and compressive stress area (blue). The average stress levels in these two areas are respectively + 4 MPa and − 2 MPa that could potentially, according to Table 4, degrade the magnetic properties. These induced mechanical stresses in the plane of the laminations allow a first analysis of the observed high level of degradation observed in the inhomogeneous case. A second point is the distribution of axial stress (${\mathbf{u}}_{\mathbf{z}}$**)** within the magnetic circuit. This stress is localized under the airvent spacers and is significant (about − 80 MPa) compared to the overall applied compaction stress (− 4 MPa). Based on the experimental measurements presented for the homogeneous case, Figs. 18 and 19, this stress level has a highly degrading effect on the global normal magnetization curve of the magnetic core.

### Effect of stress in the plane of the electrical steel sheets

The next step consists in modeling the effect of the compaction process on the magnetic properties, with and without airvent spacers. For this, it is necessary to use magneto-mechanical models that take into account mechanical stresses in the three defined directions (${\mathbf{u}}_{\mathbf{r}}$, ${\mathbf{u}}_{{\varvec{\uptheta}}}$, ${\mathbf{u}}_{\mathbf{z}}$). Having already experimentally characterized the evolution of magnetic properties as a function of axial stress (${\mathbf{u}}_{\mathbf{z}}$), Figs. 18 and 19, it is therefore necessary to characterize the effect of mechanical stress applied in the plane of the laminations. The aim is to identify the effect of stress applied in the lamination plane (in the same direction as the magnetic field) and, by relying on an equivalent stress model^18^, to be able to predict the evolution of the magnetic properties under bi-axial stress in the lamination plane.

**Figure 25:**
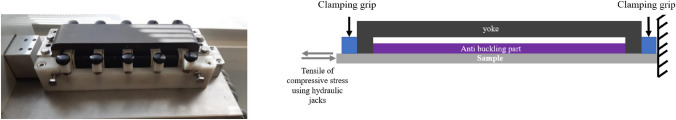
Single Sheet Tester under tensile or compression stress (left) and its schematic drawing (right).

Considering the relatively limited levels of mechanical stress (tensile and compressive) existing in the lamination plane, one can consider an approach where the bi-axial stress (along ${\mathbf{u}}_{\mathbf{r}}$ and ${\mathbf{u}}_{{\varvec{\uptheta}}}$) can be replaced by an equivalent mechanical stress that would have been applied along the magnetic flux direction. In practice, such model will require only unidirectional magneto-mechanical characterizations, i.e. the stress and the magnetic flux are in the same direction, which is realizable by conventional magneto-mechanical experimental devices. In^18^ an equivalent scalar stress model was developed and experimentally validated for the case of two-dimensional mechanical stresses applied in the plane of the laminations. This model will be further introduced in “Equivalent scalar mechanical stress and co-simulation” section. In the present work, the experimental device used to characterize the unidirectional magneto-mechanical behavior has been developed by Brockhaus Measurements and is illustrated in Fig. 25. It consists in a Single Sheet Tester where the lamination strip under test is maintained at one extremity by a fixed mechanical grip and at the other extremity by a grip actuated by an hydraulic jack. This latter allows to apply tensile or compressive stresses along the magnetic flux direction while performing the magnetic characterizations.

The magnetic measurements were performed from 5 to 300 Hz excitation frequency and from 0.1 to 1.6 T peak magnetic flux density, over the stress range [− 40 MPa; + 40 MPa]. This latter is chosen because the mechanical stresses in the lamination plane do not exceed 20 MPa for the considered compaction process, see Fig. 24a, b.

**Figure 26:**
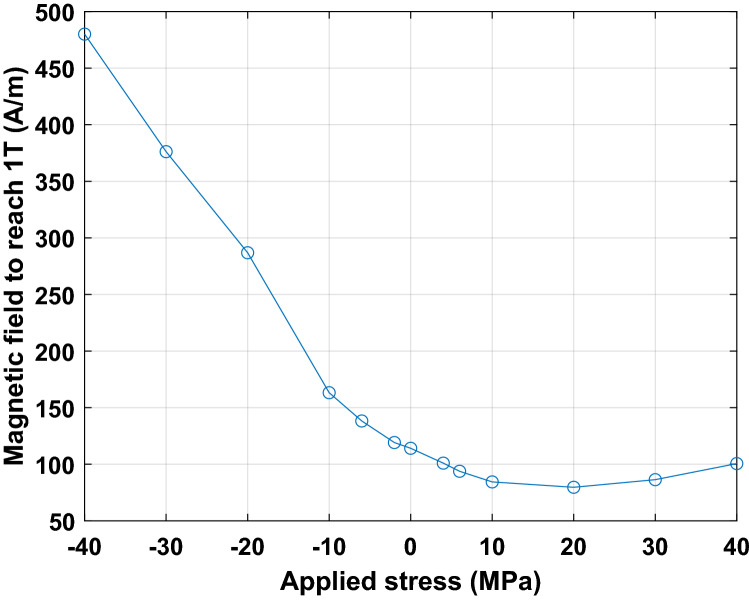
Evolution of the magnetic field necessary to reach 1 T as a function of applied mechanical stress.

**Figure 27:**
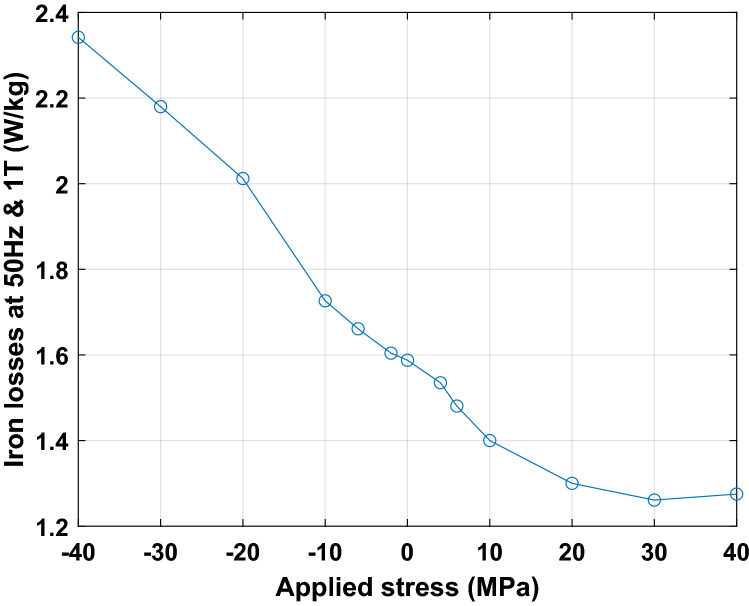
Evolution of the iron losses at 1 T as a function of applied mechanical stress.

As illustration of the measured magneto-mechanical behavior, the magnetic field and iron losses at 1T are given respectively in Figs. 26 and 27, for 50 Hz excitation frequency. These results are in accordance with the literature, Table 4, with a strong degradation of the magnetic properties under compressive stress and a slight improvement of these properties under tensile stress before observing degradation from 30 MPa.

From the results obtained for both considered configurations (axial stress only and in-plane stress only), the next step is to propose a modelling method, in the case of inhomogeneous compaction, to account for the effect of the associated three-dimensional stresses on the magnetic properties.

## Modelling

### Equivalent scalar mechanical stress and co-simulation

The equivalent scalar mechanical stress model proposed in^18^ has been experimentally validated for two-dimensional stresses in the lamination plane. However, it is theoretically independent from the geometric aspects of the considered material as it is based on an energetic approach deduced form the intrinsic magneto-mechanical properties of the material. In this model, a volume of magnetic and mechanical isotropic material is considered to calculate an equivalent magneto-elastic energy. Considering the geometry presented in the Fig. 7, the mechanical tensor ${{\varvec{\upsigma}}}_{\mathbf{m}}$, the unit vector **h,** which is along the applied magnetic flux direction, and the analytical expression of the equivalent stress ${\upsigma }_{\mathrm{eq}}$ are respectively given in (Eq. 3), (Eq. 4) and (Eq. 5). According to the literature, and to our experimental results, for mechanical stresses less than 30 MPa, if ${\upsigma }_{\mathrm{eq}}$ increases the magnetic properties are improved, if ${\upsigma }_{\mathrm{eq}}$ decreases, the magnetic properties will degrade. The term «$-\frac{1}{2}\cdot \mathrm{tr}({{\varvec{\upsigma}}}_{\mathbf{m}})$» of the (Eq. 5) implies that a compressive stress applied in an orthogonal direction (in our case ${\mathbf{u}}_{\mathbf{r}}$ and ${\mathbf{u}}_{\mathbf{z}}$) to the magnetic flux direction (in our case ${\mathbf{u}}_{{\varvec{\uptheta}}}$**)** will tend to improve the magnetic properties. Along ${\mathbf{u}}_{\mathbf{r}}$, experimental results in the literature are in accordance with this latter consideration^1^. However, our experimental results for an applied stress along ${\mathbf{u}}_{\mathbf{z}}$, Figs. 18 and 19, show that the applied compressive stress tends to degrade the magnetic properties and especially the normal magnetization curve. This means that this equivalent scalar stress model is not suitable for our case of laminar geometry. To continue the investigation and according to the complexity of implementation of an anisotropic three-dimensional magneto-mechanical model, an approach consisting in the decomposition of the effects (on the one hand, in the plane and, in the other hand, in the thickness direction) is proposed to correctly model the global effect of compaction, in the presence of airvent spacers, on the magnetic properties.

3 $${{\varvec{\upsigma}}}_{\mathbf{m}}=\left(\begin{array}{ccc}{\upsigma }_{\mathrm{rr}}& {\upsigma }_{{\mathrm{r}}{{{\uptheta}}}}& {\upsigma }_{\mathrm{rz}}\\ {\upsigma }_{{{{\uptheta}}}{\mathrm{r}}}& {\upsigma }_{{{{\uptheta}}}{{{\uptheta}}}}& {\upsigma }_{{{{\uptheta}}}{\mathrm{z}}}\\ {\upsigma }_{\mathrm{zr}}& {\upsigma }_{{\mathrm{z}}{{{\uptheta}}}}& {\upsigma }_{\mathrm{zz}}\end{array}\right)$$

4 $$h=\left(\begin{array}{c}0\\ 1\\ 0\end{array}\right)$$

5 $${\upsigma }_{\mathrm{eq}}=\frac{3}{2}\cdot {}_{ }{}^{\mathrm{t}}\mathbf{h}\cdot {{\varvec{\upsigma}}}_{\mathbf{m}}\cdot \mathbf{h}-\frac{1}{2}\cdot \mathrm{tr}({{\varvec{\upsigma}}}_{\mathbf{m}})$$

### Proposed decomposition of the effects and co-simulation protocol

Since the stresss along ${\mathbf{u}}_{\mathbf{z}}$ mainly impacts the normal magnetization curve, and according to the triaxial stress distribution, the choice is made to separate the effects of the stresses in the plane from those along the normal direction to the lamination. It is considered that the latter stress component impacts only the normal magnetization curve and that the stresses in the plane impact only the iron losses. For the normal magnetization curve, the Langevin model is used (Eq. 6) and the parameters ${\mathrm{M}}_{\mathrm{s}}$, $\mathrm{a}$ and $\mathrm{\alpha }$ are approximated by a polynomial function dependent on the stress ${\upsigma }_{{\text{z}}}$ along ${\mathbf{u}}_{{\mathbf{z}}}$.

6 $$\begin{aligned} & {\text{B}} = {\upmu }_{0} \cdot \left( {{\text{H}} + {\text{ M}}_{{{\text{an}}}} } \right) \\ & {\text{M}}_{{{\text{an}}}} = {\text{M}}_{{\text{s}}} \left( {{\upsigma }_{{\text{z}}} } \right)\left( {\coth \left( {\frac{{{\text{H}}_{{\text{e}}} }}{{{\text{a}}\left( {{\upsigma }_{{\text{z}}} } \right)}}} \right) - \frac{{{\text{a}}\left( {{\upsigma }_{{\text{z}}} } \right)}}{{{\text{H}}_{{\text{e}}} }}} \right) \\ & H_{e} = H + \alpha \left( {{\upsigma }_{{\text{z}}} } \right) \cdot M_{an} \\ \end{aligned}$$

For the iron losses, the coefficients ${\mathrm{k}}_{\mathrm{h}}$, $\mathrm{\alpha }$, ${\mathrm{k}}_{\mathrm{class}}$ and ${\mathrm{k}}_{\mathrm{exc}}$ of the loss model are also approximated by an analytical function dependent on the equivalent stress ${\upsigma }_{\mathrm{eq}}$ deduced from the plane stresses ${\upsigma }_{\mathrm{rr}}$ and ${\upsigma }_{{{{\uptheta}}}{{{\uptheta}}}}$.

### Co-simulation protocol and FE results

**Figure 28:**
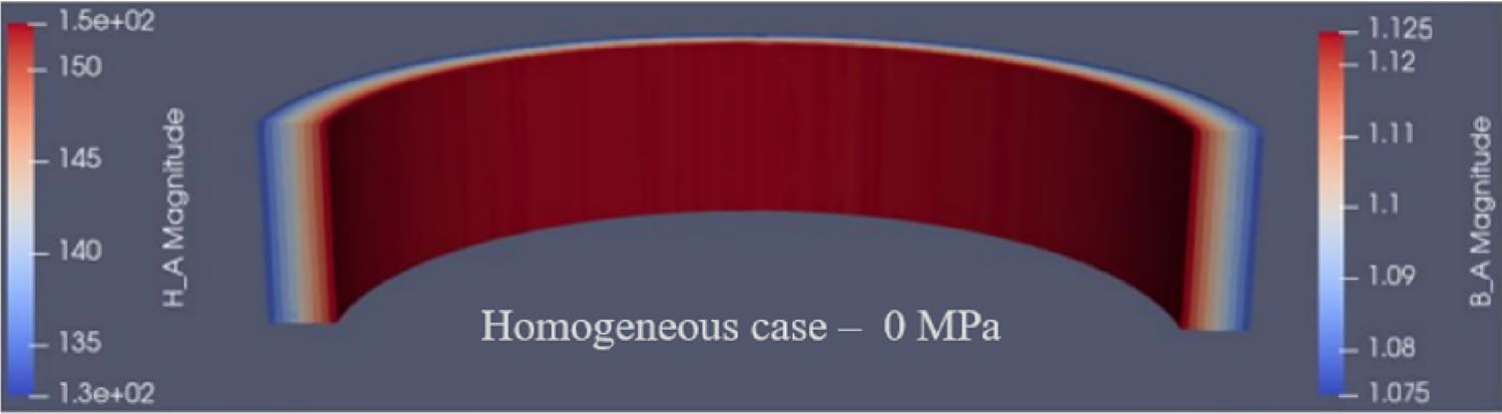
Code_Carmel simulation - Homogeneous case 0 MPa (B = 1.1 T).

**Figure 29:**
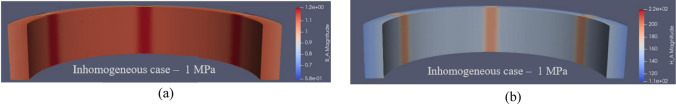
Magnetic flux density (**a**), magnetic field (**b**) Code_Carmel - Inhomogeneous 1 MPa (${\mathrm{B}}_{\mathrm{average}}$ = 1.1 T).

**Figure 30:**
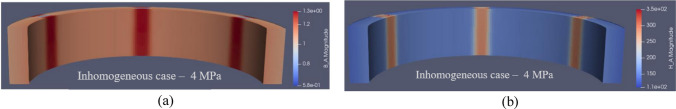
Magnetic flux density (**a**), magnetic field (**b**) Code_Carmel - Inhomogeneous 4 MPa (${\mathrm{B}}_{\mathrm{average}}$ = 1.1 T).

Subsequently, the distribution of the three-dimensional (3D) mechanical stresses presented in “Discussion” section and the associated mesh are exported from the Abaqus simulations. This mesh is then imported in Code_Carmel, a 3D Finite Element (FE) electromagnetic calculation software. To perform the simulations, the axial stress ${\upsigma }_{\mathrm{z}}$ is associated to each mesh element and the associated B-H curve is accounted for by the magneto-mechanical Langevin model also implemented in Code_Carmel. The homogeneous case is considered to validate the co-simulation protocol, then the configuration with airvent spacers presented in the paper is considered. All simulations were performed with imposed magnetic flux density in the magnetic circuit. The considered global applied stress levels are 1 MPa and 4 MPa. As illustration, the simulation results obtained for an average magnetic flux density of 1.1 T are given for the homogeneous case without applied stress in Fig. 28 and for the inhomogeneous case with applied stress in Fig. 29 (1 MPa) and Fig. 30 (4 MPa). In these FE simulations, the global magnetic flux is imposed in the cross section of the ring core leading to a local variation of the magnetic field according to the local magnetic properties that are impacted by the mechanical stress under the airvent spacers, Figs. 29 and 30. Concerning the magnetic flux density distribution, and considering that there is no leakage flux, the global magnetic flux is conserved whatever the radial cross section of the ring core. However, as the mechanical stress is mainly located under the airvent spacers, the magnetic flux will flow around this magnetically degraded area. This leads to an increased value of the magnetic flux density on both sides of the degraded area (near the inner and outer radii).

**Figure 31:**
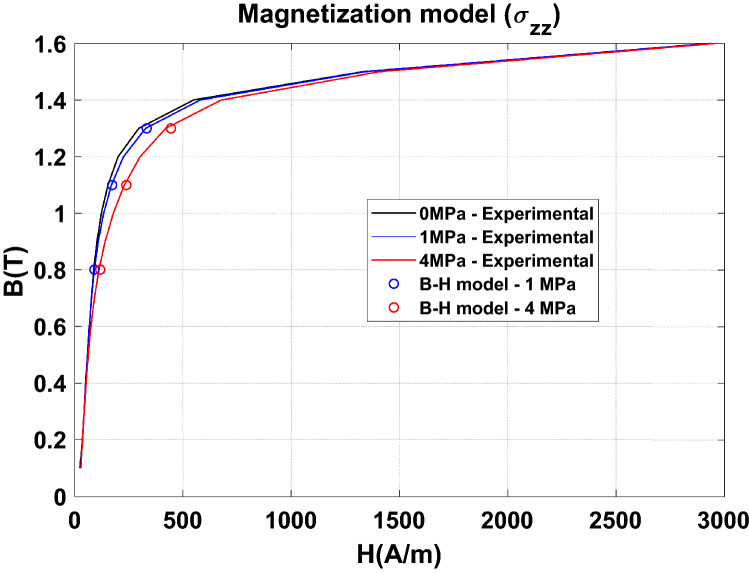
Comparison between experimental and numerical results on normal magnetization curve.

**Figure 32:**
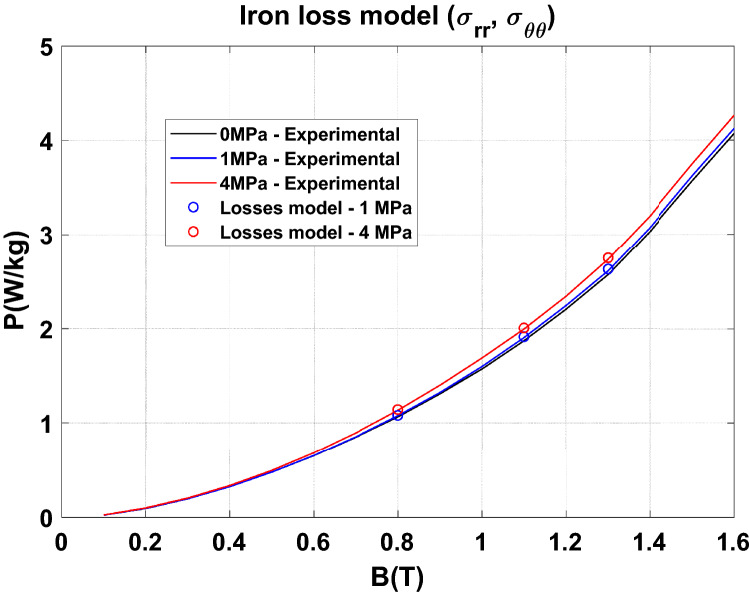
Comparison between experimental and numerical results on iron losses.

From the magnetic flux density distribution, the iron losses are calculated in post-processing with the magneto-mechanical loss model taking into account the equivalent stress in the plane of the laminations. Results in terms of magnetic field and iron losses, considering a magnetic flux density of 1.1 T, are given in Table 5. They show that the proposed approach provides a good approximation of the compaction process effect on the magnetic properties. In particular, this emphasizes that the axial stress (${\mathbf{u}}_{\mathbf{z}}$) is mainly detrimental to the normal magnetization curve and that the stresses in the plane (${\mathbf{u}}_{\mathbf{r}},$ ${\mathbf{u}}_{\mathbf{z}}$) are impactful for the iron losses. The results are extended to three levels of magnetic flux density in Figs. 31 and 32 (white circles) demonstrating the same conclusions.

**Table 5:** Effect of the heterogeneous compaction on the magnetic field and iron losses at 1.1 T.

Global compaction stress	Magnetic field at 1.1 T		Iron losses at 1.1 T	
	Experimental	Model (${\upsigma }_{zz})$	Experimental	Model (${\upsigma }_{\mathrm{rr}}$,${\upsigma }_{{{{\uptheta}}}{{{\uptheta}}}})$
1 MPa	+ 11%	+ 13%	+ 1.9%	+ 2.3%
4 MPa	+ 50%	+ 55%	+ 6.5%	+ 7.2%

Comparison between the experimental and simulation results—the relative difference (in %) is given compared to the case without compaction.

### Discussion

One can consider that the proposed approach is a good qualitative approximation of the impact on magnetic properties of the compaction process with very satisfactory results despite the assumptions. These latter are especially based on the decoupling of in-plane stresses from those in the thickness direction of which effects are strongly non-linear. Second, characterizations for the axial stress applied in the homogeneous case were performed up to 20 MPa, so the normal magnetization curve model was identified over this same range of stress values. In the inhomogeneous case, for a global stress of 1 MPa, the maximum ${\upsigma }_{zz}$ stresses that appear locally are of the order of 20 MPa. However, for a global applied stress of 4 MPa, this maximum stress value reaches 80 MPa. For a stress level beyond 20 MPa, the B-H model was therefore applied in a stress region where the magnetization behavior is extrapolated, which represents a strong hypothesis. It should also be noted that if no extrapolation has been made for the iron losses (because the in-plane stress levels were within the identification range of the model), their calculation is obviously influenced by the accuracy of the normal magnetization curve modeling, which means that an overestimation of the effect on the latter can induce an overestimation of the effect on iron losses. For all these reasons and in order to confirm our results, a simple approach consists in performing the calculation by swapping the input data in the materials models (behavior law and iron losses) between the axial and in-plane stresses. In practice, the normal magnetization curve was dependent only on the equivalent stress determined from the stress distribution in the lamination plane whereas the iron losses were dependent only on the axial stress. For this, the models have been identified according to the same protocol previously presented. The normal magnetization curve model considering ${\upsigma }_{\mathrm{rr}}$ and ${\upsigma }_{{{{\uptheta}}}{{{\uptheta}}}}$ was implemented in Code_Carmel and the iron losses calculated in post-processing from the magnetic flux density distribution and the associated stress map ${\upsigma }_{\mathrm{zz}}$. Results are given in Table 6, showing that neglected stresses in the first simulation configuration (Table 5) do indeed have a negligible impact on the magnetic properties.

**Table 6:** Effect of the heterogeneous compaction on the magnetic field and iron losses at 1.1 T.

Global compaction stress	Magnetic field at 1.1 T		Iron losses at 1.1 T	
	Experimental	Model (${\upsigma }_{\mathrm{rr}}$,${\upsigma }_{{{{\uptheta}}}{{{\uptheta}}}})$	Experimental	Model (${\upsigma }_{zz})$
1 MPa	+ 11%	+ 0.7%	+ 1.9%	+ 0%
4 MPa	+ 50%	+ 5%	+ 6.5%	+ 0.5%

Configuration with swapped input stresses in the material models.

Finally, it must be highlighted that since the effect of stress in the plane of the laminations and in the axial direction were characterized separately, it was implicitly assumed that their effects were superposable, which is not necessarily the case due to the complex coupled phenomena involved in the magneto-mechanical behavior of electrical steels. However, the proposed approach remains quite useful for assessing the global impact of the heterogeneous compaction of electrical steel laminations as commonly performed for high power electrical machines.

## Conclusions and perspectives

The presented work deals with the experimental study of the effect of an industrial compaction process on magnetic cores performances. This study was associated to the numerical modeling of the impact of this process. In particular, an experimental device with a capacity of 5 tons has been developed in order to apply a compaction effort with and without airvent spacers while being able to both control the mechanical stress distribution but also characterize the magnetic properties. The significant degradation effect induced by the presence of airvent spacers has been demonstrated as well as, for the first time, the particular effect of homogeneous compaction on magnetic properties. Indeed, concerning the latter, a significant deterioration of the normal magnetization curve, mainly in the saturation knee, is observed as well as a very limited effect on the iron losses, which is quite different from what is commonly observed when applying mechanical stresses in the plane of laminations. The physical origins of the behavior observed during compaction along ${\mathbf{u}}_{\mathbf{z}}$ are currently difficult to define. The fact of having a significant degradation on the normal magnetization curve and not on the iron losses can be explained by the appearance of a potential energy that hinders the domain walls motion in the saturation-knee region. Moreover, this result opens up new perspectives for the understanding of the magneto-mechanical behavior of electrical steel laminations for axial mechanical stresses.

Nevertheless, an approach to model the effect of compaction was proposed and validated taking into account the three-dimensional mechanical stresses present in the magnetic circuit by separating the axial stresses from the ones in the plane of the laminations. If this approach is valid in the case of compaction, it should be possible to work on a three-dimensional magneto-mechanical model. Finally, the effect of compaction and the developed modeling approach can be studied on more complex geometries such as the stator of electrical machines.

## Data Availability

The datasets used and/or analyzed during the current study are available from the corresponding author on reasonable request.
